# Primary Microcephaly Gene *MCPH1* Shows Signatures of Tumor Suppressors and Is Regulated by miR-27a in Oral Squamous Cell Carcinoma

**DOI:** 10.1371/journal.pone.0054643

**Published:** 2013-03-05

**Authors:** Thejaswini Venkatesh, Mathighatta Nagaraj Nagashri, Shivananda S. Swamy, S. M. Azeem Mohiyuddin, Kodaganur S. Gopinath, Arun Kumar

**Affiliations:** 1 Department of Molecular Reproduction, Development and Genetics, Indian Institute of Science, Bangalore, Karnataka, India; 2 Department of Surgical Oncology, Bangalore Institute of Oncology, Bangalore, Karnataka, India; 3 Department of Otolaryngology and Head and Neck Surgery, R. L. Jalappa Hospital and Research Centre, Kolar, Karnataka, India; University of Navarra, Spain

## Abstract

Mutations in the *MCPH1* (microcephalin 1) gene, located at chromosome 8p23.1, result in two autosomal recessive disorders: primary microcephaly and premature chromosome condensation syndrome. MCPH1 has also been shown to be downregulated in breast, prostate and ovarian cancers, and mutated in 1/10 breast and 5/41 endometrial tumors, suggesting that it could also function as a tumor suppressor (TS) gene. To test the possibility of *MCPH1* as a TS gene, we first performed LOH study in a panel of 81 matched normal oral tissues and oral squamous cell carcinoma (OSCC) samples, and observed that 14/71 (19.72%) informative samples showed LOH, a hallmark of TS genes. Three protein truncating mutations were identified in 1/15 OSCC samples and 2/5 cancer cell lines. MCPH1 was downregulated at both the transcript and protein levels in 21/41 (51.22%) and 19/25 (76%) OSCC samples respectively. A low level of *MCPH1* promoter methylation was also observed in 4/40 (10%) tumor samples. We further observed that overexpression of MCPH1 decreased cellular proliferation, anchorage-independent growth in soft agar, cell invasion and tumor size in nude mice, indicating its tumor suppressive function. Using bioinformatic approaches and luciferase assay, we showed that the 3′-UTR of *MCPH1* harbors two non-overlapping functional seed regions for miR-27a which negatively regulated its level. The expression level of miR-27a negatively correlated with the MCPH1 protein level in OSCC. Our study indicates for the first time that, in addition to its role in brain development, *MCPH1* also functions as a tumor suppressor gene and is regulated by miR-27a.

## Introduction

The *MCPH1* (microcephalin 1) gene, also known as *BRIT1* (BRCT-repeat inhibitor of TERT expression 1), is located at chromosome 8p23.1. It was initially recognized as an inhibitor of human telomerase in an ERM (enhanced retroviral mutagens) screen [1]. Homozygous mutations in this gene cause an autosomal recessive disorder, primary microcephaly (MCPH), which is characterized by decreased size of cerebral cortex and mental retardation in affected individuals [2]. A homozygous mutation in this gene also causes another autosomal recessive disorder, premature chromosome condensation (PCC) syndrome [3]. *MCPH1* consists of 14 coding exons and codes for an 835 amino acids long protein of 110 kDa. It is widely expressed in different tissues, including the brain [2]. MCPH1 harbors three BRCT (BRCA1 C-terminal) domains, a NLS (nuclear localization signal) and a CIIBR (condensin II binding region). It belongs to the BRCT family of proteins that are involved in DNA repair [2], [4]. In response to DNA damage, MCPH1 recruits ATM, MDC1 and NBS1 to DNA damage repair foci in U2OS (osteosarcoma) cells [4]. It remodels the chromatin by interacting with SWI-SNF complex during DNA repair [5], and mediates homologous repair by interacting with NCAPG2 subunit of condensin II [6]. It also interacts with E2F1 and positively regulates the expression of pro-apoptotic genes such as *BRCA1*, *CHEK1*, *TP73*, *CASP3* and *CASP7* in U2OS cells [7]. Also, the knockdown of MCPH1 in HEK293 cells downregulates the level of *BRCA1* transcript [7]. *MCPH1* codes for a centrosomal protein and partially targets CHEK1 to centrosomes [8], [9]. Depletion of MCPH1 leads to ionizing radiation induced centrosome amplification by dysregulation of CHEK1-controlled CDK2 activation in DT40 chicken B cells [10]. Deficiency of MCPH1 causes PCC by dysregulation of CHEK1 mediated activation of centrosomal CCNB1-CDK1 complex in U2OS and MCPH1 null lymphoblastoid cells [9]. The knockout mouse models of MCPH1 show deficiency in DNA repair, premature chromosome condensation and defective spindle orientation [11], [12], [13].

Microsatellite analysis has previously shown LOH (loss of heterozygosity) at the D8S518 and D8S277 markers flanking the MCPH1 locus in 1/21 oral tumors [14]. Lu et al. [15] have observed LOH at the D8S1742 and D8S277 markers flanking the MCPH1 locus in 2/32 hepatocellular carcinomas. A 38 bp homozygous deletion in *MCPH1* was also reported in 1/10 breast tumors [4]. Bilbao et al. [16] screened the *MCPH1* gene for mutations within mononucleotide coding tracts in exons 4, 5 and 8 in 41 MSI (microsatellite instability)-positive and 62 MSI-negative endometrial tumors and found mutations in only five MSI-positive tumors. Most of these mutations were in a heterozygous state [16]. Further, MCPH1 was found to be downregulated at the transcript level in 19/30 ovarian cancer specimens and at the protein level in 93/319 breast cancer tissues [4], [17]. Decreased MCPH1 protein levels are associated with triple negative breast cancers and a lower transcript level of *MCPH1* correlates with lesser time for metastasis in breast cancer [4], [17]. Interestingly, MCPH1 knockout mice in a null TP53 background show susceptibility to cancers [11]. However, MCPH1 knockout mouse models or the microcephaly patients exhibit no susceptibility to cancers [11], [12], [13]. Based on these observations, we hypothesized that *MCPH1* may also function as a tumor suppressor (TS) gene, in addition to its role in the brain development. The purpose of this study was to test if MCPH1 also functions as a TS gene using different approaches in OSCC (oral squamous cell carcinoma). TS genes show some or all of the following signatures: LOH, somatic mutations, promoter methylation, downregulated expression in tumors and reduced cell proliferation upon overexpression. The results of our study show that *MCPH1* has many of these signatures, functions as a TS gene and is regulated by miR-27a.

## Materials and Methods

### Ethics Statement

This research followed the tenets of the Declaration of Helsinki, and the informed written consent for research was obtained from the patients enrolled in the study following the approval from the ethics committee of the Bangalore Institute of Oncology. The nude mice experiments were approved by the Animal Ethics Committee of the Indian Institute of Science.

### Sample Collection

A total of 93 OSCC patients were included in our study (Tables S1 and S2 in File S1). Of these, two patients had epithelial dysplasia (ED). The surgically resected tissues from oral cancerous lesions and normal tissues taken from the farthest margin of surgical resection were obtained from the Bangalore Institute of Oncology, Bangalore. Tissues were collected in RNA Later™ (Sigma-Aldrich, St Louis, MO) immediately after surgery. Peripheral blood samples were also collected in EDTA-Vacutainer™ tubes (Becton-Dickinson, Franklin Lakes, NJ). The tumors were classified according to the TNM (Tumor, Node and Metastasis) criteria [18]. The details of clinico-pathological features of the patients are shown in the Table S2 in File S1.

### Cell Lines

A549 (human lung adenocarcinoma), HeLa (human cervical carcinoma) and KB (oral squamous cell carcinoma) cell lines were procured from the National Repository for cell lines at National Centre for Cell Sciences, Pune, India. The KB cell line has been described to be contaminated with HeLa cells by the American Type Culture Collection (ATCC, Manassas, VA). Oral squamous cell carcinoma cell lines, SCC084 (UPCI:SCC084) and SCC131 (UPCI:SCC131), were a kind gift from Dr. Susanne M. Gollin (University of Pittsburgh, Pittsburgh, PA). These cell lines were generated from consenting patients undergoing surgery for squamous cell carcinoma of the oral cavity at the University of Pittsburgh Medical Center, following Institutional Review Board guidelines [19]. Cell lines were maintained in Dulbecco’s modified Eagle’s medium (DMEM) supplemented with 10% FBS and 1X antibiotic-antimycotic mix (Sigma-Aldrich, St Louis, MO).

### Genomic DNA Isolation

Genomic DNA samples from blood, cell lines and tissue samples were isolated using either the Flexigene® DNA Isolation kit (Qiagen, Valencia, CA) or the Wizard® Genomic DNA purification kit (Promega, Madison, WI) according to the manufacturers’ instructions.

### LOH Analysis

For LOH analysis, 81 matched normal and tumor DNA samples were genotyped using D8S1819, D8S277 and D8S1798 markers flanking the MCPH1 locus as described in Kumar et al. [20]. Briefly, the forward primer of each marker was first radiolabelled using γ-^32^P-ATP (3,000 ci/mmole; BRIT, Hyderabad, India) and T_4_ PNK (Bangalore Genei®, Bangalore, India). PCR was then carried out in a PTC-100 thermal cycler (MJ Research Inc., Waltham, MA) with radiolabelled forward primer and cold reverse primer using a standard PCR protocol. Radiolabelled PCR products were resolved in a sequencing gel, transferred to a Whatman™# 1 filter paper, wrapped in a thin plastic sheet, dried and scanned using a FLA 2000 Phosphor Image System (Fuji, Tokyo, Japan). In order to detect LOH in a tumor sample, band intensities of larger and smaller alleles of a marker were quantitated using the Alpha DigiDoc 1201 software (Alpha InfoTech Corporation, San Leandro, CA) and expressed as integrated density values. The LOH index was calculated as the ratio of the intensity of the larger allele to the smaller allele of the tumor divided by the same of its corresponding blood/normal oral tissue. The LOH index of <0.65 and >1.5 was considered as LOH for smaller and larger alleles respectively [21].

### Mutation Analysis

For mutation analysis, the entire coding region and intron-exon junctions of the *MCPH1* gene were amplified with 17 primers pairs (Table S3 in File S1). Primers were designed using the gene sequence retrieved from the National Centre for Biotechnology Information web site (http://www.ncbi.nlm.nih.gov/). PCR was carried out in a 25 µl reaction volume using a PTC-100 thermocycler under the following conditions: an initial denaturation at 95°C for 2 min was followed by denaturation at 95°C for 30 sec, annealing at 55–64°C for 30 sec and extension at 72°C for 1 min for 35 cycles with a final extension at 72°C for 5 min. Following amplification, PCR products were run on 2% agarose gels containing ethidium bromide, eluted from the gels using the GenElute™ Extraction Kit (Sigma-Aldrich, St Louis, MO) and sequenced on an ABIprism A370-automated sequencer (PE Biosystems, Foster City, CA). Mutations were confirmed by sequencing both DNA strands.

### Western Blotting

Total protein lysates from tissues and cell lines were prepared using the CelLytic™ MT Mammalian Tissue Lysis and Extraction Reagent, and the CellLytic™ M Cell Lysis Reagent (Sigma-Aldrich, St Louis, MO), respectively, according to the manufacturer’s instructions. Protein lysates were resolved on an 8% low cross-linking SDS-polyacrylamide gel and Western blotting was performed as described earlier [22]. Briefly, the Western blot was incubated with a polyclonal anti-MCPH1 antibody (cat# ab2612) from Abcam (Cambridge, MA) or Sigma-Aldrich (cat# HPA008238) at a dilution of 1∶500. The signal on the blot was detected using an HRP-conjugated secondary antibody (Bangalore Genei®, Bangalore, India), Immobilon Western chemiluminescent HRP substrate (Millipore, Darmstadt, Germany) and X-ray films (Kodak, Rochester, NY).

### RNA Isolation and First-strand cDNA Synthesis

Total RNA was isolated from ∼100 mg of the tissue sample using the TRI REAGENT™ (Sigma-Aldrich, St Louis, MO). The first-strand cDNA was synthesized from 1.5 µg of total RNA using the Revert Aid™ H Minus First-Strand cDNA Synthesis Kit (MBI Fermentas, Burlington, ON) according to the manufacturer’s instructions.

### Quantitative Real-time PCR

Quantitative real-time PCR was performed using the Dynamo SYBR® green mix (Finnzymes, Espoo, Finland) in an ABIprism H7000 sequence detection system and analyzed using the SDS 2.1 software (Applied Biosystems, Foster City, CA). *Glyceraldehyde-3-phosphate dehydrogenase* (*GAPDH*) was used as a normalizing control [23, Table S4 in File S1]. For PCR, primers were designed on two different exons to rule out the possibility of amplification of contaminating genomic DNA. The GenBank accession numbers for *MCPH1*, *GAPDH* and *BRCA1* are BC030702, BC001601 and JN686490.1 respectively. Primer sequences and their PCR conditions are detailed in Table S4 in File S1. The relative expression of *MCPH1* was calculated by 2^–ΔΔCt^ method as described by Livak and Schmittgen [24]. To accommodate the unpaired tumor samples, we calculated the relative expression in a tumor as 2^−ΔCt (tumor)^/2^−ΔCt (mean of normal tissues)^. The relative expression was categorized into downregulated, upregulated or no change according to 50%–150% cut-off levels [25]. The 2^−ΔΔCt^ method was also used to analyze the relative expression of *MCPH1* in a set of 24 matched normal and OSCC samples. However in this case, the relative expression in a tumor was calculated as 2^−ΔCt (tumor)^/2^−ΔCt (matched normal)^. The significance of difference in the expression levels between tumor and normal tissue samples was determined by the paired Student’s t-test. The relative expression of *BRCA1* in 24 matched normal and OSCC samples was calculated as described for *MCPH1*.

### Immunohistochemistry

The immunohistochemistry for the tissue slides was performed as described previously by Simmons et al. [26] using a rabbit polyclonal anti-MCPH1 antibody (*Abcam,* Cambridge, MA) at a dilution of 1∶100 and a secondary antibody from Bangalore Genei®, Bangalore, India. The degree of MCPH1 staining in tumors was scored as the percentage of cells stained with DAB (3,3-diaminobenzidine) in a total of five microscopic fields at a magnification of 40X. A 35% cut-off was used to dichotomise the MCPH1 expression into low and high groups as described by Richardson et al. [17].

### Promoter Methylation Analysis

The *MCPH1* promoter sequence was retrieved from the Transcriptional Regulatory Element Database (TRED) (http://rulai.cshl.edu/TRED). The *MCPH1* promoter (ID: 39666) was examined for the presence of CpG islands using the CpG plot/CpG Report software (http://www.ebi.ac.uk/emboss/cpgplot). To examine the methylation status of CpG islands, sodium bisulfite treatment of genomic DNA and COBRA were carried out as described previously [27]. For COBRA, CpG island I (CpGI) and CpG island II (CpGII) were amplified by nested PCR. Primers sequences and their PCR conditions are detailed in Table S4 in File S1. Following amplification, PCR products were resolved in a 2% agarose gel, eluted from the gel and digested with either *Bst* UI (MBI Fermentas, Burlington, ON) for CpGI or *Aci* I (MBI Fermentas, Burlington, ON) for CpGII. As a positive control to test if the enzymes are active, a pair of primers (Table S4 in File S1) were designed to amplify a 356 bp fragment of the exon 1 of the *ASPM* gene which harbors three sites for each of *Bst* UI and *Aci* I. In some cases, the CpGI and CpGII amplicons were cloned into the pTZ57R TA cloning vector (MBI Fermentas, Burlington, ON). TA clones were then purified using the GenElute™ Plasmid Miniprep kit (Sigma-Aldrich, St Louis, MO) and sequenced as described above to identify methylation status of CpG sites.

### Treatment of Cells with *2*′-*deoxy*-*5*-*azacytidine*

Cells were treated with *2*′-*deoxy*-*5*-*azacytidine* (AZA) (Sigma-Aldrich, St Louis, MO) to see the effect of methylation on the expression of *MCPH1* as described in Bajaj et al. [27]. Semi-quantitative RT-PCR was performed to determine the levels of *MCPH1*. *ß-actin* was used as a normalizing control (Table S4 in File S1). For each gene, PCR was optimized to obtain amplification in a linear range. Following electrophoresis of the PCR products on 2% agarose gels containing ethidium bromide, the images were captured with the UltraCam™ Digital imaging system (Bangalore Genei®, Bangalore, India). The band intensities were quantitated as described above using the Alpha DigiDoc 1201 software. The relative expression is plotted as the ratio of integrated density values of the amplicon of *MCPH1* to that of *ß-actin*. The Student’s t-test was used to determine statistical significance.

### Generation of Stable Clones

The pcDNA3.1(+)/*MCPH1*/myc-His construct containing a full-length *MCPH1* was a kind gift from Prof. A. Jackson, MRC Human Genetics Unit, Edinburgh, UK. The empty vector pcDNA3.1(+)/myc-His was a kind gift from Prof. P. Kondaiah, Indian Institute of Science, Bangalore, India. All the transfections were performed with Lipofectamine™ 2000 (Invitrogen, Carsland, CA) according to the manufacturer’s instructions. For stable transfections, 1 µg of pcDNA3.1(+)/*MCPH1*/myc-His or pcDNA3.1(+)/myc-His was transfected separately into KB cells and after a recovery period of 48 hours, cells were maintained in DMEM containing 1 mg/ml G418 (Calbiochem, San Diego, CA) for two weeks. Single clones were then picked, expanded in culture and screened by Western blotting as described above for MCPH1 level using an anti-MCPH1 antibody.

### Cell Proliferation Assay

The cell proliferation assay was performed using the CHEMICON® BrdU Cell Proliferation Assay Kit (Milipore Corporation, Billerica, MA). Briefly, 5,000 cells were seeded per well of a 96-well plate and after 2 days, they were incubated with BrdU for 20 hours, fixed for 30 min and then incubated overnight with a BrdU detection antibody. Cells were washed and incubated in the goat anti-mouse-IgG, peroxidase labeled solution. Following washing, cells were incubated with the substrate, and the plate was read at 450 nm using a BIO-RAD™ microplate reader (BIO-RAD™ Laboratories, Hercules, CA). The experiment was repeated thrice independently. The graph was plotted with optical density (OD) values. The statistical significance was determined by one-way ANOVA.

### Soft Agar Colony Assay

The soft agar colony assay was performed as described by Baumann Kubetzko et al. [28]. Briefly, 5,000 cells from KB and other stable clones were allowed to grow separately for 21 days in soft agar and then stained with 0.05% crystal violet (Sigma-Aldrich, St Louis, MO). The colonies were photographed using an Olympus CKX41 phase contrast microscope (Olympus Optical Co., Tokyo, Japan). The number of colonies were counted in five random microscopic fields at a magnification of 10X. The statistical significance was determined by one-way ANOVA.

### *In vivo* Tumorigenicity Assay in Nude Mice

The *in vivo* tumorigenicity assay of stable clones with empty vector pcDNA3.1(+)/myc-His or *MCPH1* overexpression was performed in 4–6 weeks old BALB/c nude mice as described by Tsai et al. [29]. Cells from the stable clones V2, V4, B1 and B9 were injected separately into the right flanks of nude mice and the tumor volume was measured at regular intervals using a digital Vernier’s caliper. The volume of the tumor was given as ½ (width^2^ × length). The xenografts harvested on 42^nd^ day were weighed, fixed in 5% formaline, sectioned and stained with hemotoxylin and eosin (Nice Chemicals Pvt Ltd, Kochi, India). The statistical analysis was carried out using unpaired Student’s t-test.

### Propidium Iodide Staining for Cell Death

To assess the levels of apoptosis, 1×10^6^ cells were resuspended in 200 µl of DPBS (Sigma-Aldrich, St Louis, MO) and fixed with 70% ethanol overnight at 4°C. Cells were then treated with 60 µg of RNAase A (Bangalore Genei®, Bangalore, India) overnight at 37°C and incubated with 50 µg of propidium iodide (Sigma-Aldrich, St Louis, MO) for 20 min on ice. Cells were shielded from light and immediately analyzed by flow cytometry on a FACSCalibur™ (BD Biosciences, San Jose, CA). All experiments were repeated thrice independently. The graph was plotted for the percentage of cells in sub-G1, G1/S, S and G2/M phases.

### Analysis of CASP3 Activity for Apoptosis

The level of apoptosis in cells was measured with the *CaspGLOW*™ Fluorescein Active Caspase Staining *Kit* (Bio Vision Inc., San Francisco, CA) according to the manufacturer’s instructions. Briefly, 0.3 million cells were incubated with FITC conjugated CASP3 inhibitor and then analyzed for FITC signal by flow cytometry. All experiments were repeated thrice independently. The graph was plotted for the activity of CASP3 and the statistical significance was determined by one-way ANOVA.

### Cell Invasion Assay

The cell invasion assay was performed using a BD BioCoat™ Matrigel™ Invasion Chamber (BD Biosciences, San Jose, CA) according to the manufacturer’s instructions. Briefly, 7.5×10^4^ cells prepared in DMEM containing 2% FBS were plated in the upper chamber (transwell) of the matrigel-coated membrane. The lower chamber was filled with 0.75 ml of DMEM containing 5% FBS as a chemoattractant. Transwells were incubated for 22 hours at 37°C. After incubation, cells on the inside of the transwell inserts were removed with a cotton swab, and the cells on the underside of the insert filter were fixed with methanol and stained with 0.05% crystal violet. Cells were photographed and counted in four random microscopic fields to calculate the number of cells that had invaded through the matrigel matrix. All experiments were repeated thrice independently. The graph was plotted for the number of cells that had invaded per microscopic field, and the significance of difference in invasiveness between two clones was determined by one-way ANOVA.

### *In silico* Analysis of MicroRNA Targets in *MCPH1*

The 5.4 kb 3′-UTR of *MCPH1* was analyzed for miRNA binding sites using five miRNA prediction softwares: MicroCosm (http://www.ebi.ac.uk/enright-srv/microcosm), miRTAR (http://mirtar.mbc.nctu.edu.tw/), microRNA (http://www.microrna.org/microrna/home.do), miRDB (http://mirdb.org/miRDB/) and TargetScan (http://www.targetscan.org/).

### Generation of Constructs for miRNA Studies

In order to generate a construct with a full-length pre-miR-27a, amplification was carried out using specific primers (Table S5 in File S1) and human genomic DNA as a template. PCR product was then cloned in the TA cloning vector pTZ57R (MBI Fermentas, Burlington, ON). After verifying the insert by sequencing, the insert was released by double digestion of the TA clone with *Hind* III (MBI Fermentas, Burlington, ON) and *Xho* I (MBI Fermentas, Burlington, ON) and subcloned in the pcDNA3-*EGFP* vector. Similarly, the seed regions 1 and 2 separately as well as together in sense and antisense orientations were amplified using specific primers (Table S5 in File S1), and the amplicons were first cloned in the TA cloning vector. After verifying the TA clones by sequencing, the inserts were released by digestion with *Sac* I (MBI Fermentas, Burlington, ON) and *Hind* III and subcloned into the pMIR-Report vector (Ambion, Austin, TX). PCR conditions for these primers are given in Table S5 in File S1.

### Site-directed Mutagenesis

The site-directed mutagenesis in the seed regions 1 (75 to 81 nt) and 2 (5383 to 5389 nt) was carried out as described in Wang and Malcolm [30]. Seed regions 1 and 2 cloned separately and together in the pTZ57R TA cloning vector were used as templates for site-directed mutagenesis using primers given in Table S5 in File S1. Mutations in TA clones were confirmed by sequencing, and the inserts were then subcloned into the pMIR-Report vector using *Hind* III and *Sac* I restriction sites.

### Luciferase Reporter Assay

The interaction between 3′-UTR of *MCPH1* and miR-27a was determined using a Dual-Luciferase® Reporter Assay System (Promega, Madison, WI) according to the manufacturer’s instructions. For Luciferase assay, 2×10^4^ KB cells were seeded per well in a 24-well plate one day prior to transfection. Different combination of vector and constructs were then co-transfected in KB cells. The pRL-TK vector containing *Renilla* luciferase gene was also co-transfected as an internal control to normalize for transfection efficiency. Luciferase and Renilla activities were measured using a TD-20/20 Luminometer (Turner Designs, Sunnyvale, CA) after two days of transfection. All the co-transfections were performed in triplicates for each transfection experiment, and the whole experiment was repeated independently thrice. The luciferase activity was calculated in relative luciferase units (RLU) and the statistical significance was determined using unpaired Student’s t-test.

### Semi-quantitative RT-PCR of miR-27a

Total RNA samples from tissues or cells were converted to cDNA using a miR-27a specific primer RT6-miR-27a (Table S4 in File S1) and the Revert Aid™ H Minus First-Strand cDNA Synthesis Kit according to the manufacturer’s instructions. For semi-quantitative RT-PCR, miR-27a was amplified in a reaction mix containing short-miR-27a, MP-fw and MP-rev primers using a standard PCR protocol (Table S4 in File S1). The *5S rRNA* was used as a normalizing control (Table S4 in File S1). Primer sequences for miR-27a, MP-fw (universal primer), MP-rev (universal primer) and *5S rRNA* were obtained from Sharbati-Tehrani et al. [31].

### Statistical Analysis

All statistical analyses were performed using the GraphPad Prism 5 software (GraphPad Prism Software, San Diego, CA). Fischer’s exact test was used to correlate the expression levels of MCPH1 transcript or protein with the clinico-pathological parameters such as gender of the patients, T stages of the tumors, age of the patients and site of the tumors. p<0.05 was considered to be significant. A correlation between the transcript levels of *MCPH1* and *BRCA1* was determined using the Pearson’s correlation coefficient.

## Results

### LOH at the MCPH1 Locus

**Figure 1:**
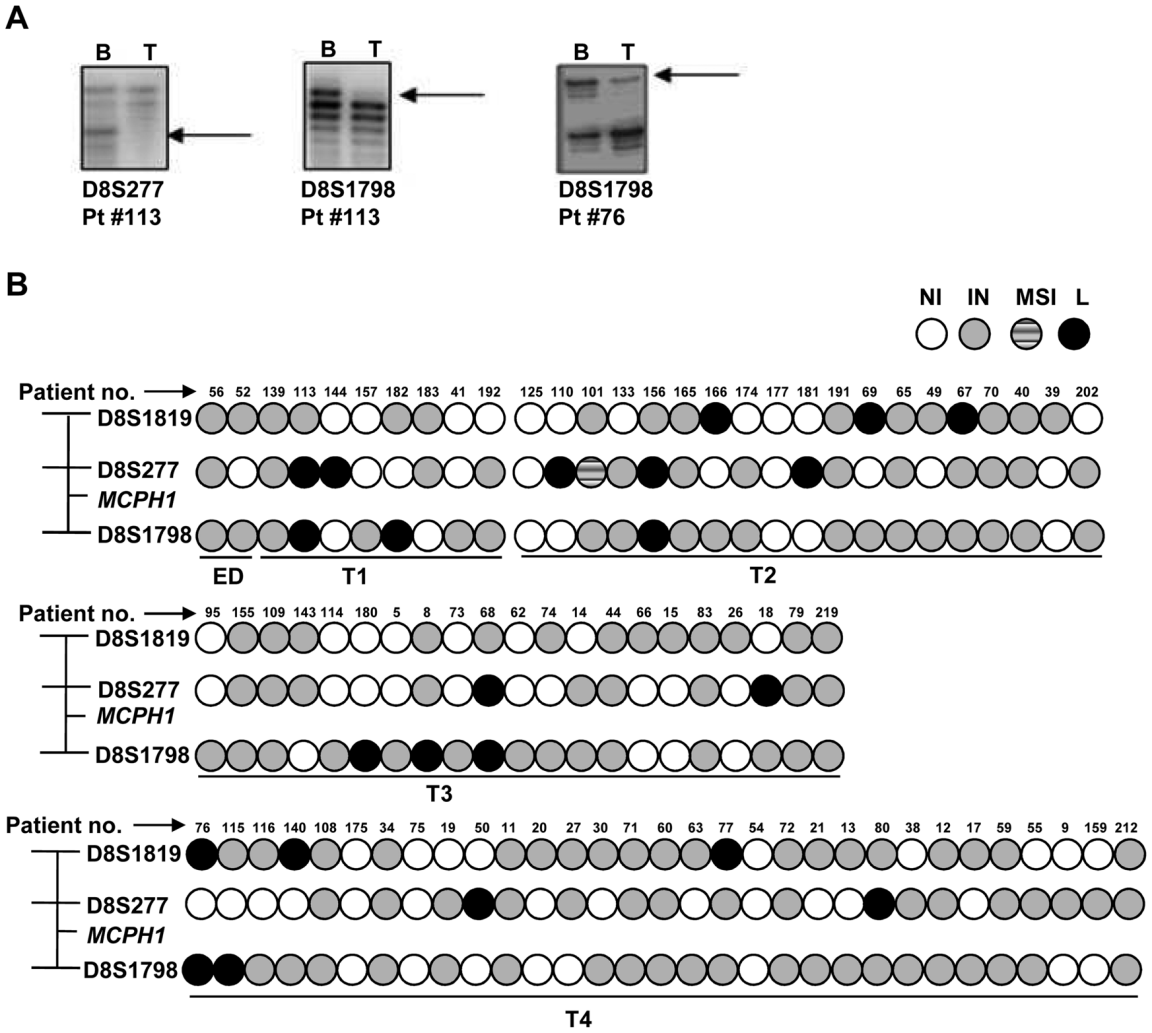
LOH at the MCPH1 locus. (A) Representative phosphor images showing LOH for the markers D8S1819, D8S277 and D8S1798 flanking the MCPH1 locus. B & T denote constitutive blood/normal oral tissue and tumor DNA respectively. Arrows indicate the loss of alleles in tumor DNA. (B) Diagrammatic representation of LOH data from 81 matched blood/normal oral tissue and tumor DNA samples using three microsatellite markers. Tumor samples are arranged according to their T classification (T1 to T4) or ED (epithelial dysplasia). Abbreviations: NI, non-informative; IN, informative; MSI, microsatellite instability; and, L, LOH.

In order to see if the region harboring the *MCPH1* gene shows LOH in OSCC samples, we performed LOH study in a panel of 81 matched blood/normal oral and tumor tissues using three microsatellite markers (viz., D8S1819, D8S277 and D8S1798) flanking the MCPH1 locus. The results showed LOH at one or more markers in 14/71 (19.72%) informative samples across the tumor stages from T1 to T4 (Figure 1).

### Mutations in *MCPH1*

**Figure 2:**
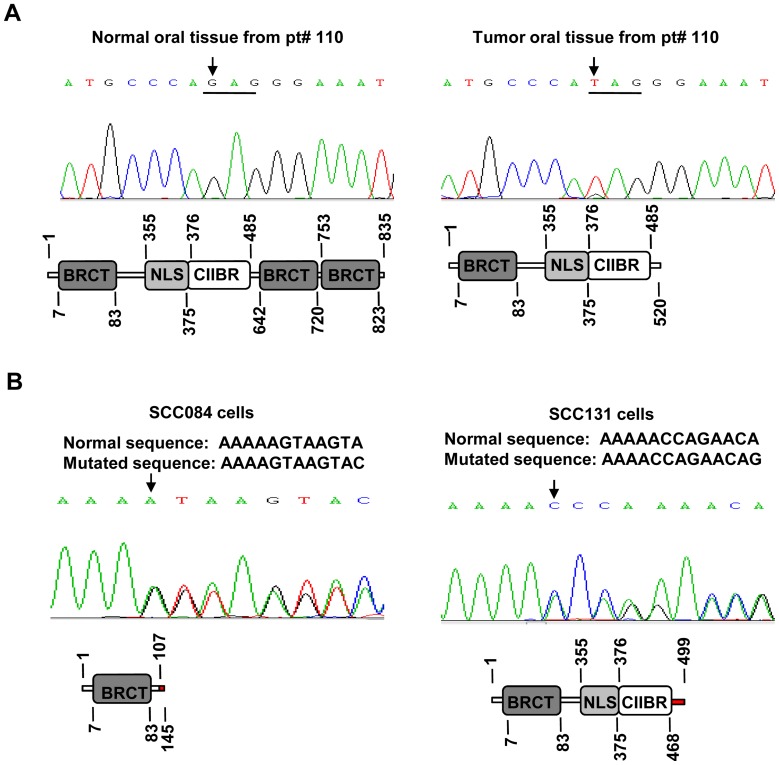
Mutations in the *MCPH1* gene. (A) Sequencing chromatograms from the normal and tumor oral tissues of the pt# 110 with a nonsense mutation c.1561G>T (p.Glu521X) in a homozygous state. The LOH study has shown deletion of one allele in this tumor (Figure S1 in File S2), suggesting that both alleles are non-functional due to either deletion or mutation. Arrows mark the sites of the mutation in chromatograms. The codon affected by the mutation is underlined. Schematic representations of wild-type and mutant MCPH1 proteins are shown below the chromatograms. The wild-type protein is 835 amino acids long and harbors three BRCT domains, a NLS (nuclear localization signal) and a CIIBR (condensin II binding region). The truncated mutant protein is predicted to be 520 amino acids long with the loss of two C-terminal BRCT domains. (B) Sequencing chromatograms from SCC084 and SCC131 cells with the mutations c.321delA (p.Lys107fsX39) in exon 4 and c.1402delA (p.Thr468fsX32) in exon 8, respectively, in a heterozygous state. Schematic representations of the mutant proteins are shown below the chromatograms. Red bars in protein structure represent abnormal amino acids.

As TS genes show somatic mutations in tumor samples, the entire coding region and intron-exon junctions of the *MCPH1* gene were sequenced in 15 OSCC samples and five cancer cell lines (viz., A549, HeLa, KB, SCC084 and SCC131). Only one of the 15 OSCC samples, pt# 110, showed a somatic truncating mutation c.1561G>T(p.Glu521X) in exon 8 in a homozygous state (Figure 2A). Interestingly, pt# 110 had also shown LOH in the tumor tissue (Figure S1 in File S2), suggesting that one of the alleles is mutated and the other one is deleted in this tumor sample. Two OSCC cell lines SCC084 and SCC131 showed frame-shift mutations c.321delA(p.Lys107fsX39) in exon 4 and c.1402delA(p.Thr468fsX32) in exon 8, respectively, in a heterozygous state (Figure 2B). No mutations were identified in A549, HeLa and KB cells.

To examine if the mutations are affecting the MCPH1 level in SCC084 and SCC131 cells, we used Western blot analysis. The results showed that the MCPH1 level was relatively lower in mutated cell lines SCC084 and SCC131 in comparison to the non-mutated cell line KB (Figure S2 in File S2). Although, A549 and HeLa cells did not harbour any mutation and yet showed a lower level of MCPH1 in comparison to KB cells, suggesting the involvement of an alternative mechanism for its regulation in these cells (Figure S2 in File S2).

### Downregulation of MCPH1

Since TS genes are expected to show downregulation in a majority of the tumor samples, if not in all, we sought to determine the levels of MCPH1 transcript and protein in OSCC samples. Real-time quantitative RT-PCR of 41 OSCC samples and 24 normal oral tissues showed downregulation of *MCPH1* transcript in 21/41 (51.22%) and upregulation in 7/41 (17.07%) tumors in comparison to the mean expression value of 24 normal tissues (Figure S3 in File S2). There was no change in its expression in 13/41 (31.71%) tumors in comparison to the mean expression value of 24 normal oral tissues (Figure S3 in File S2). Out of 41 OSCC samples, 24 had their corresponding matched normal oral tissues. These 24 matched normal and tumor samples were considered as a separate set and the relative expression of *MCPH1* in tumors was compared with that in matched normal tissues. Of 24 paired samples, it was downregulated in 13 (54.16%) tumors, upregulated in 3 (12.5%) tumors, and no change in its expression was observed between eight matched normal and tumor samples (Figure S4 in File S2).

We also performed immunohistochemistry to examine its protein levels in 25 matched OSCC samples. MCPH1 was expressed in both nucleus and cytoplasm across all the oral tumor and normal tissue samples. All normal tissues exhibited moderately strong staining of MCPH1. The highest expression of MCPH1 was seen in the epithelial regions, followed by the muscular regions. Using 35% cut-off, the MCPH1 expression was low and high in 19/25 (76%) and 6/25 (24%) tumors, respectively (Figure S5 in File S2). The above observations suggested that MCPH1 is downregulated in a majority of the OSCC samples both at the transcript and protein levels.

### Promoter Methylation of *MCPH1*

**Figure 3:**
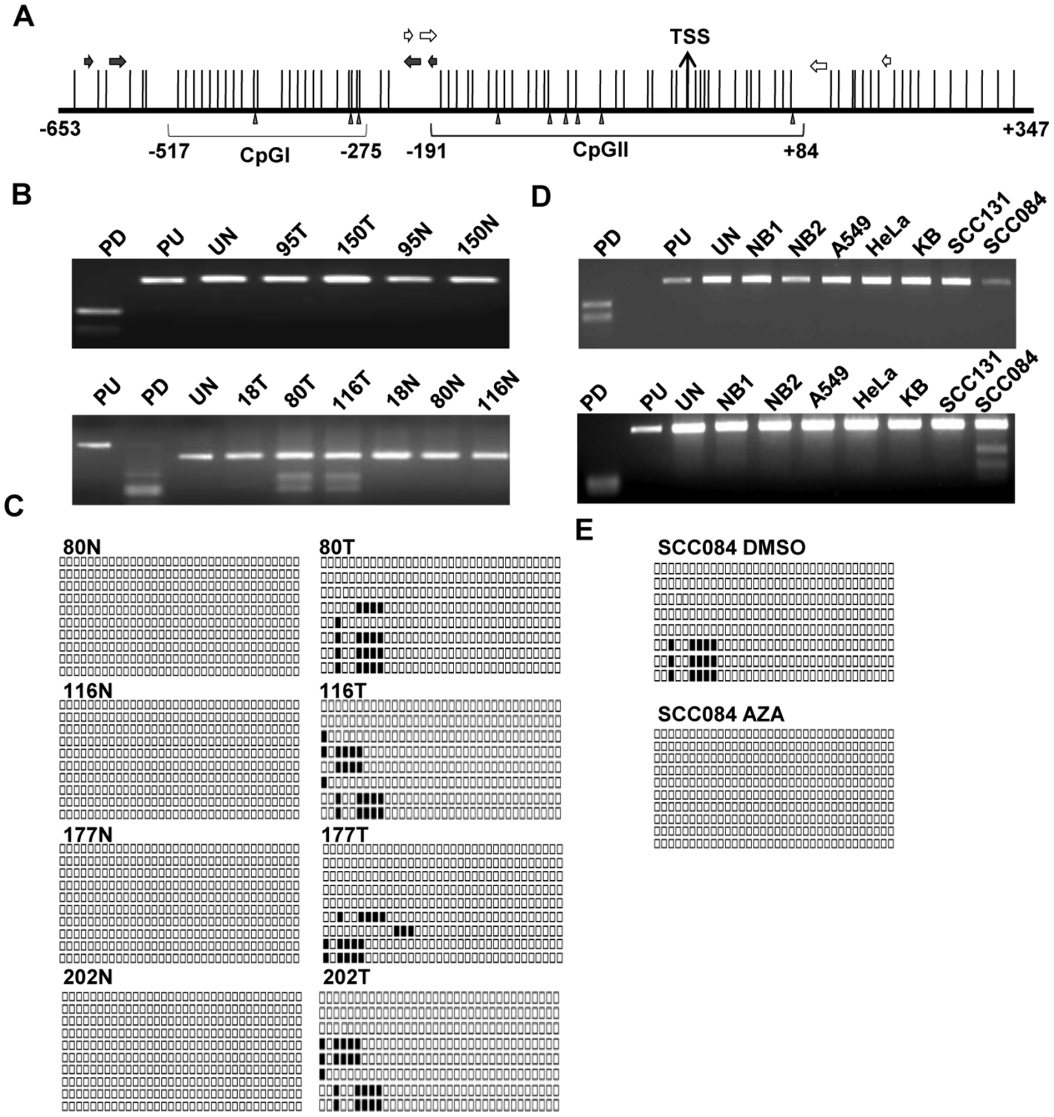
Methylation of CpG islands in the *MCPH1* promoter. (A) Schematic representation of the *MCPH1* promoter with two CpG islands. The vertical lines represent the CpG sites. The solid and open horizontal arrows represent primers to amplify CpGI and CpGII islands respectively. Sites for *Bst* UI and *Aci* I in CpGI and CpGII, respectively, are marked by filled vertical arrowheads. The numbers represent nucleotide positions with respect to the TSS. (B) Representative agarose gel images of COBRA for CpGI (upper panel) and CpGII (lower panel). Note the absence of methylation of CpGI in tumor samples 95T and 150T, and the methylation of CpGII in tumor samples 80T and 116T. (C) Schematic representation of bisulfite treated genomic DNA sequence of CpGII in normal and tumor tissues from patient numbers 80, 116, 177 and 202. Each row represents a sequenced TA clone. The filled and unfilled squares represent methylated and unmethylated CpGs respectively. Note the methylation of tumor samples and non-methylation of their corresponding normal oral tissues. (D) Representative agarose gel images of COBRA data for CpGI (upper panel) and CpGII (lower panel) in cell lines. None of the cell lines show CpGI methylation, whereas CpGII shows methylation in SCC084 cells only. (E) Bisulfite sequencing of CpGII in SCC084 cells before and after the AZA (2′-deoxy-5-azacytidines) treatment. The CpG sites in CpGII show methylation in DMSO (vehicle control) treated DNA, whereas, as expected, methylation is lost after AZA treatment. Abbreviations: N, normal; T, tumor; PD, positive control (*ASPM* fragment); PU, positive control undigested; UN, undigested CpG island I or II; and, NB1 or NB2, peripheral blood DNA from unrelated normal individuals. Numbers represent patient numbers.

Since methylation of promoters has been found in many TS genes as a mechanism for their downregulation, we wanted to investigate if the promoter of *MCPH1* is methylated in OSCC samples. In order to study if its promoter is methylated, we first retrieved the *MCPH1* promoter using bioinformatics analysis. The *MCPH1* promoter (ID: 39666) lies between -653 bp to +347 bp relative to transcription start site (TSS) and harbors two CpG islands, CpGI and CpGII (Figure 3A and Figure S6 in File S2). Since methylation is observed in CpG islands, we then used COBRA technique to examine their methylation status in a panel of 40 OSCC samples. The CpGI was found to be negative for methylation (Figure 3B, upper panel). Whereas, the CpGII showed methylation in a small number of 4/40 OSCC samples (viz., pt# 80, 116, 177 and 202) (Figure 3B, lower panel). As expected, their corresponding normal oral tissues were negative for CpGII methylation (Figure 3B, lower panel). Further, these four matched normal and tumor tissue samples were sequenced to verify the COBRA data. As anticipated, no methylation of CpGII was observed in normal tissues, whereas their corresponding tumors showed methylation (Figure 3C and Figure S7 in File S2). These observations suggested that the *MCPH1* promoter is methylated in tumors, although at a low level. This also suggested that the promoter methylation is not a major mechanism of its downregulation in tumors.

To further examine the relationship between the promoter methylation and downregulation (transcription) of *MCPH1*, we treated HeLa, KB, SCC084 and SCC131 cells with 2′-deoxy-5-azacytidine (AZA), a known methylation transferase inhibitor, for 3 and 5 days and determined its expression by semi-quantitative RT-PCR. No change in its expression was observed in HeLa and KB cells, whereas the expression was increased in SCC084 and SCC131 cells following the treatment (Figure S8 in File S2), suggesting methylation of its promoter. We then analyzed the methylation of CpGI and CpGII in these cells by COBRA. None of the cell lines showed methylation in CpGI (Figure 3D, upper panel), whereas the results showed methylation of CpGII in SCC084 cells only (Figure 3D, lower panel). We then sequenced the CpGII region in SCC084 cells using sodium bisulfite treated DNA before and after the AZA treatment for 5 days. As expected, the CpGII was methylated in SCC084 cells prior to the treatment and its methylation was lost after the treatment (Figure 3E and Figure S9 in File S2). This suggested that CpGII methylation is responsible for the downregulation of *MCPH1* in SCC084 cells. CpGII sequences did not show methylation in the rest of the cells lines (viz., HeLa, KB and SCC131) without the AZA treatment (Figure S10 in File S2) and therefore CpGII methylation was not studied in these cell lines after the treatment. Additionally, we sequenced CpGI in KB, SCC084 and SCC131 cells, and one OSCC sample (pt# 150) before the AZA treatment using sodium bisulfite treated DNA. As expected, no methylation was observed in all these samples (Figure S11 in File S2).

### MCPH1 Reduces Cell Proliferation

**Figure 4:**
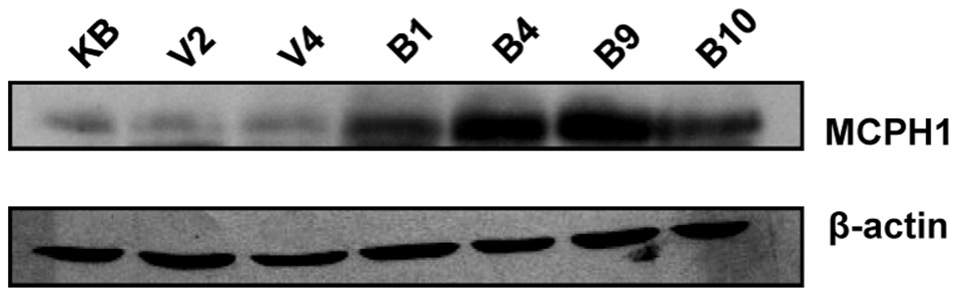
MCPH1 overexpression in stable clones. Representative images of Western blots of the lysates from stable clones generated with either the pcDNA3.1(+)/*MCPH1*/myc-His construct or the empty vector pcDNA3.1(+)/myc-His after G418 selection in KB cells. ß–actin was used as a loading control. Abbreviations: KB, parental cell line; V2 and V4, stable clones with the vector only; and, B1, B4, B9 and B10, MCPH1 overexpressing stable clones.

**Figure 5:**
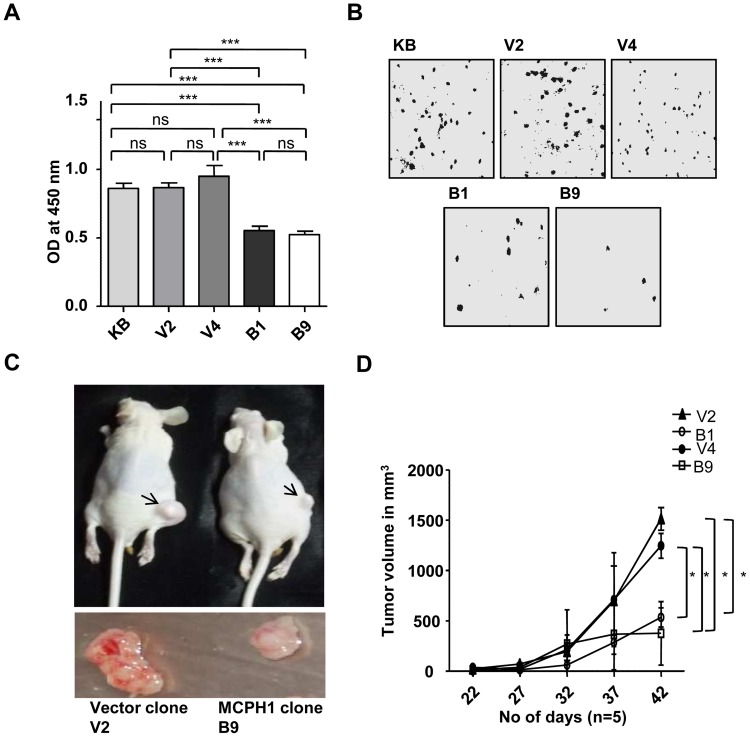
Tumor suppressor activity of MCPH1. (A) The analysis of cell proliferation in KB (parental cell line), empty vector stable clones (V2 and V4) and MCPH1 overexpressing stable clones (B1 and B9) by the BrdU assay. The graph represents the OD (optical density) measurment at 450 nm. Note the B1 and B9 clones incorporated significantly lesser BrdU than the V2, V4 and KB cells. The values are the mean±SD of three independent experiments. Abbreviations: ns, no statistical significance; and, *** indicates p<0.001. (B) Anchorage-independent growth of KB, V2, V4, B1 and B9 cells in soft agar. Note MCPH1 overexpression reduces colony growth of B1 and B9 clones as compared to KB, V2 and V4 cells. (C) The *in vivo* tumorigenic potential of V2, V4, B1 and B9 cells in nude mice. The representative images of the nude mice with tumors (upper panel) and the harvested tumors (lower panel) are shown. Note that the overexpression of MCPH1 reduces tumor growth in nude mice. The arrows mark the tumors. (D) Tumor volumes at different time points. The values given are the mean±SD from five animals. * indicates p<0.05.

TS genes reduce cell proliferation. In order to test if MCPH1 reduces cell proliferation, we generated four MCPH1 overexpressing stable clones (viz., B1, B4, B9 and B10) in KB cells by G418 selection (Figure 4). Henceforth, we have used two MCPH1 overexpressing clones B1 and B9 with moderate to high overexpression in all the experiments. Two stable clones (viz., V2 and V4) harboring the empty vector (pcDNA3.1(+)/myc-His) were also generated in KB cells as controls (Figure 4). To understand the effect of MCPH1 on cell proliferation, we cultured the cells from KB, V2, V4, B1 and B9, and the level of BrdU incorporation was measured after 20 hours of BrdU treatment. The results showed increased incorporation of BrdU in KB, V2 and V4 cells in comparison to B1 and B9 cells, suggesting that MCPH1 reduces cellular proliferation (Figure 5A).

### MCPH1 Inhibits Growth of Colonies in Soft Agar

We also performed the soft agar colony forming assay to see the effect of MCPH1 overexpression on inhibition of anchorage-independent growth of colonies. The results showed a significant decrease in number of colonies in MCPH1 overexpressing B1 and B9 clones as compared to KB cells and, V2 and V4 clones, suggesting its tumor suppressive function (Figure 5B and Figure S12 in File S2).

### MCPH1 Suppresses Tumor Growth in Nude Mice

The observation of decreased cell proliferation and growth of colonies in soft agar of B1 and B9 cells prompted us to study the tumor suppressive function of MCPH1 in nude mice. The results showed that the volumes of xenografts from V2 and V4 cells were significantly higher than those from B1 and B9 cells (Figure 5C, D). Similarly, the tumor weights of V2 and V4 cells on day 42 were significantly higher than those of B1 and B9 clones (Figure S13 in File S2). The visual observation of tumor regression on *MCPH1* overexpression was further validated by examining the paler necrotic regions within hematoxylin and eosin stained histological sections of the xenografts. The results showed higher necrotic regions in MCPH1 overexpressing xenografts than the vector xenografts (Figure S14 in File S2). These observations suggested that MCPH1 also suppresses tumor growth in nude mice.

### MCPH1 Induces Apoptosis

**Figure 6:**
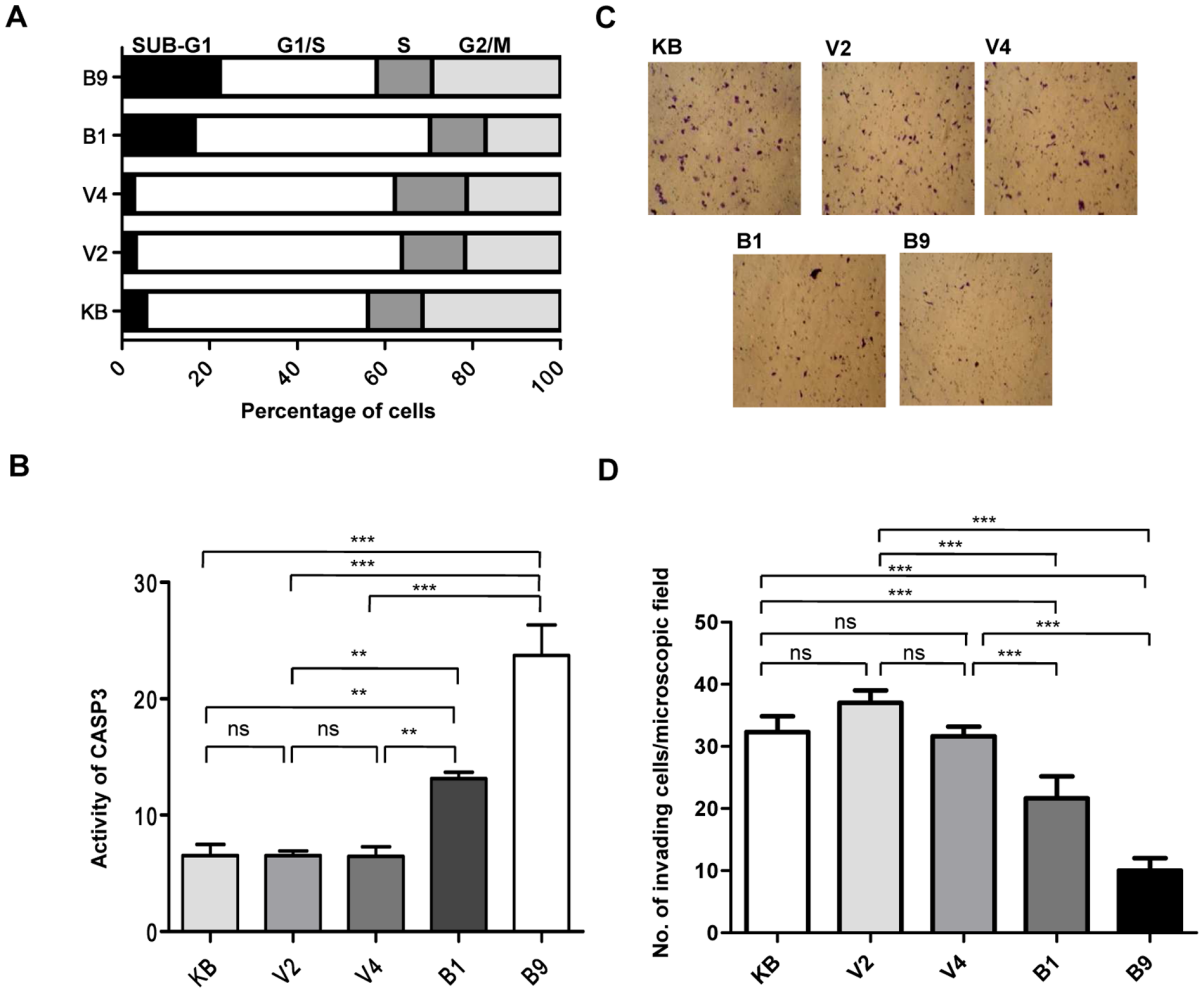
Increased apoptosis and decreased invasion in MCPH1 overexpressing cells. (A) The analysis of sub-G1 populations (a measure of cell death) in PI stained KB, V2, V4, B1 and B9 cells by flow cytometry. The graph represents one of the three separate experiments. Note higher sub-G1 populations in MCPH1 overexpressing B1 and B9 cells in comparison to KB, V2 and V4 cells. The mean values (%) ± SDs for sub-G1 populations in different cells are as follows: KB, 5.07±0.63; V2, 2.06±0.71; V4, 2.40±0.48; B1, 16.16±0.57; and B9, 22.64±0.45. (B) Analysis of apoptosis by *in* *vitro* quantitation of CASP3 activity. Note significantly increased CASP3 activity in B1 and B9 cells in comparison to KB, V2 and V4 cells. The values shown are mean±SD of three separate experiments. (C) Analysis of cell invasion in KB cells, and V2, V4, B1 and B9 clones. Note *MCPH1* overexpression reduced invasiveness in B1 and B9 cells as compared to KB, V2 and V4 cells. (D) The quantitative representation of the cell invasion assay data. The values are the mean±SD of the number of invaded cells counted in four random microscopic fields. Abbreviations: ns, statistically not significant; **p<0.005; and, ***p<0.001.

We were further interested in understanding the mechanism of tumor suppression by MCPH1. We stained KB, V2, V4, B1 and B9 cells with PI (propidium iodide) and quantitated the proportion of apoptotic cells in sub-G1 stage by flow cytometry. The proportion of sub-G1 cells in B1 and B9 cells was considerably higher than in KB, V2 and V4 cells (Figure 6A), suggesting that MCPH1 induces cell death.

To determine the type of cell death, we then performed a more sensitive assay such as the activation of CASP3 to assess the levels of apoptosis in stables clones. The results showed a significantly higher CASP3 activity in B1 and B9 cells than in KB, V2 and V4 cells, suggesting that MCPH1 induces apoptosis by upregulating CASP3 activity (Figure 6B and Figure S15 in File S2).

### *MCPH1* Inhibits Cell Invasion

We performed the cell invasion assay to see the effect of *MCPH1* overexpression on the invasive ability of KB cells. The results showed a significant decrease in number of cells that had invaded through the matrigel matrix in *MCPH1* overexpressing B1 and B9 clones as compared to KB cells and, V2 and V4 clones (Figure 6C, D).

### miR-27a Targets *MCPH1*

**Figure 7:**
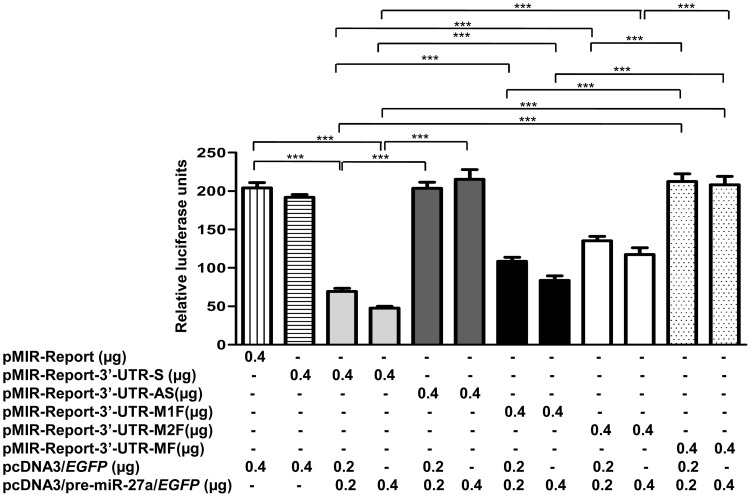
miR-27a targets both the seed regions of *MCPH1* cloned together. The luciferase reporter assay of different constructs of the full-length 3′-UTR of *MCPH1* in KB cells. Note a significantly reduced luciferase activity in cells co-transfected with pMIR-Report-3′-UTR-S and pcDNA3/pre-miR-27a/*EGFP* (0.2 or 0.4 µg) in comparison to those with pMIR-Report or pMIR-Report-3′-UTR-AS, suggesting miR-27a targets *MCPH1*. Note that both the seed regions in the 3′-UTR of *MCPH1* are important for the interaction with miR-27a as a significant increase in the luciferase activity was observed in cells transfected with pMIR-Report-3′-UTR-M1F, pMIR-Report-3′-UTR-M2F or pMIR-Report-3′-UTR-MF along with pcDNA3/pre-miR-27a/*EGFP* in comparison to cells transfected with pMIR-Report-3′-UTR-S and pcDNA3/pre-miR-27a/*EGFP.* Abbreviations: ns, statistically not significant; and, ***p<0.001.

The low rates of LOH, mutation and promoter methylation of *MCPH1* in OSCC samples suggested that they may not be the major mechanisms for the downregulation of *MCPH1*. Since miRNAs have recently been shown to post-transcriptionally regulate genes, we sought to determine if *MCPH1* is regulated by miRNAs. We then used five miRNA prediction softwares and identified miR-27a and miR-27b as potential miRNAs which could target and regulate *MCPH1* (Table S6 in File S1). Henceforth, we chose miR-27a for further analysis. The 5.4 kb 3′-UTR of *MCPH1* contains two miR-27a seed regions (SDRs): seed region 1 (75–81 nt) and seed region 2 (5383–5389 nt) (Figure S16 in File S2). We cloned them together as well as separately into the pMIR-Report vector. To validate if miR-27a targets *MCPH1*, we co-transfected the pMIR-Report-3′-UTR-S construct containing both the seed regions (full-length 3′-UTR) of *MCPH1* in a sense orientation and the pcDNA3/pre-miR-27a/*EGFP* construct containing the pre-miR-27a in KB cells. As a control, we transfected the pMIR-Report vector in KB cells. As a second control, we also co-transfected the pMIR-Report-3′-UTR-AS construct containing the full-length 3′-UTR region of *MCPH1* in an antisense orientation and the pcDNA3/pre-miR-27a/*EGFP*. After 2 days, the cells were lysed and the luciferase activity was measured. As compared to both the controls, the luciferase activity was significantly decreased in cells co-transfected with pMIR-Report-3′-UTR-S and pcDNA3/pre-miR-27a/*EGFP*, suggesting that miR-27a negatively regulates *MCPH1* level (Figure 7). To determine if both or only one of the seed regions are binding to miR-27a, we mutated each of the two SDRs by site-directed mutagenesis (Table S5 in File S1). We then co-transfected the pMIR-Report-3′-UTR-M1F construct containing the mutated SDR1 and the wild-type SDR2, pMIR-Report-3′-UTR-M2F construct containing the wild-type SDR1 and the mutated SDR2 or pMIR-Report-3′-UTR-MF construct containing both the mutated SDR1 and SDR2 separately in KB cells along with pcDNA3/pre-miR-27a/*EGFP*. The results showed a significant increase in the luciferase activity in cells transfected with pMIR-Report-3′-UTR-MF and pcDNA3/pre-miR-27a/*EGFP* in comparison to cells transfected with pMIR-Report-3′-UTR-S and pcDNA3/pre-miR-27a/*EGFP* (Figure 7). Further, the luciferase activity was significantly higher in cell transfected with the double mutant pMIR-Report-3′-UTR-MF and pcDNA3/pre-miR-27a/*EGFP* in comparison to cells transfected with single mutants pMIR-Report-3′-UTR-M1F or pMIR-Report-3′-UTR-M2F with pcDNA3/pre-miR-27a/*EGFP* (Figure 7). Further, the luciferase activity was significantly decreased in cells transfected with pMIR-Report-3′-UTR-S and pcDNA3/pre-miR-27a/*EGFP* in comparison to cells transfected single mutants, pMIR-Report-3′-UTR-M1F or pMIR-Report-3′-UTR-M2F and pcDNA3/pre-miR-27a/*EGFP* (Figure 7). These observations suggest that both SDRs bind to miR-27a.

**Figure 8:**
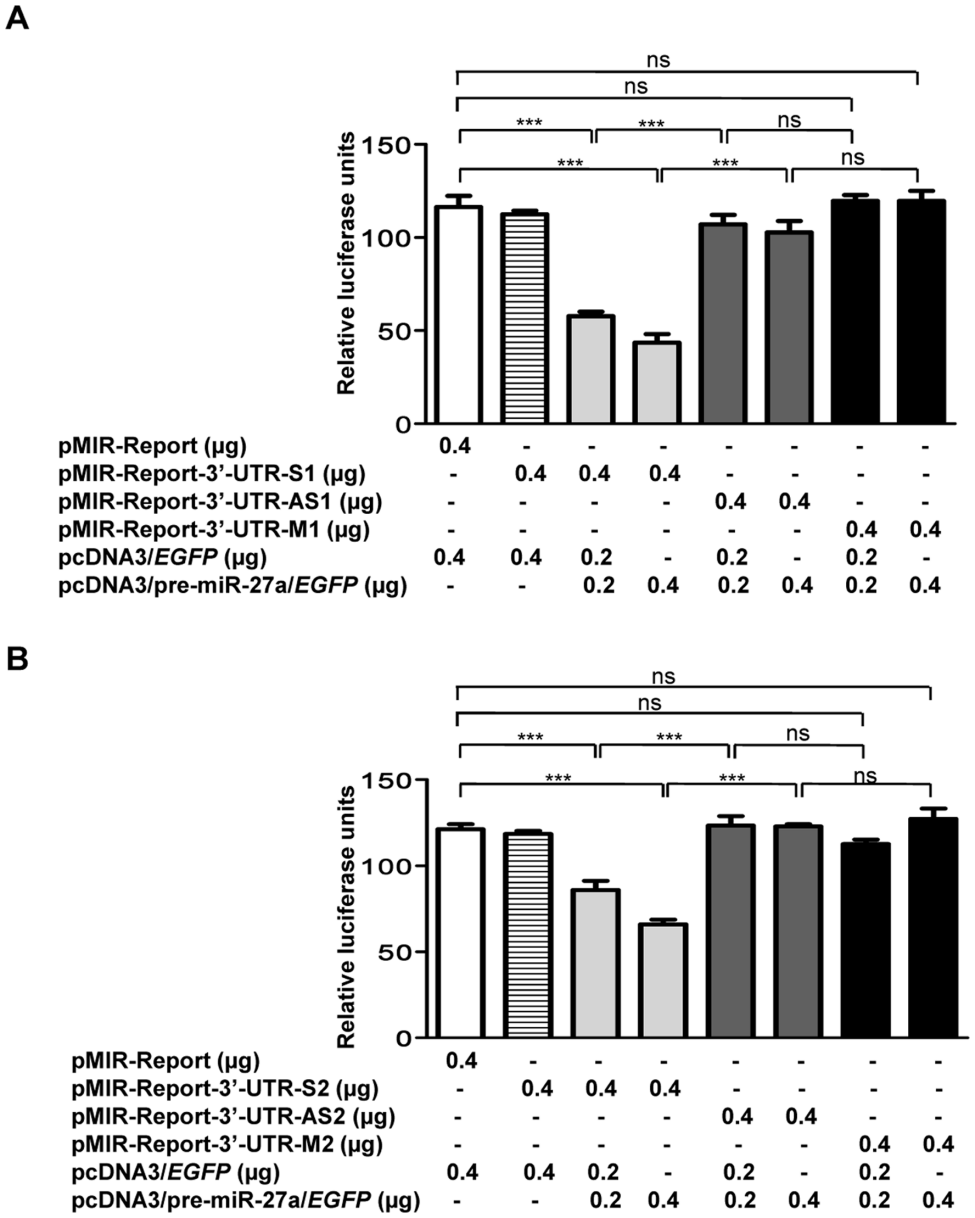
miR-27a targets both the seed regions of *MCPH1* cloned separately. **(**A) The luciferase reporter assay of different constructs harboring seed region 1 (SDR1) of *MCPH1* 3′-UTR in KB cells. Note a significantly reduced luciferase activity in cells co-transfected with pMIR-Report-3′-UTR-S1 and pcDNA3/pre-miR-27a/*EGFP* (0.2 or 0.4 µg) in comparison to those with pMIR-Report, suggesting miR-27a targets SDR1 of *MCPH1*. As expected, no significant difference in luciferase activity was observed in cells co-transfected with pMIR-Report-3′-UTR-AS1 or pMIR-Report-3′-UTR-M1 with pcDNA3/pre-miR-27a/*EGFP* (0.2 or 0.4 µg) in comparison to those with pMIR-Report. (B) The luciferase reporter assay of different constructs harboring seed region 2 (SDR2) of *MCPH1* 3′-UTR in KB cells. As with SDR1, miR-27a also targets SDR2.

We also cloned both the seed regions separately in the pMIR-Report vector and co-transfected the pMIR-Report-3′-UTR-S1 construct containing seed region 1 (SDR1) of *MCPH1* in a sense orientation and the pcDNA3/pre-miR-27a/*EGFP* construct in KB cells. As a control, we transfected the pMIR-Report vector in KB cells. As a second control, we also co-transfected the pMIR-Report-3′-UTR-AS1 containing SDR1 of *MCPH1* in an antisense orientation and the pcDNA3/pre-miR-27a/*EGFP*. After 2 days, the cells were lysed and the luciferase activity was measured. As compared to controls, the luciferase activity was decreased in cells co-transfected with pMIR-Report-3′-UTR-S1 and pcDNA3/pre-miR-27a/*EGFP*, suggesting that miR-27a negatively regulates MCPH1 level (Figure 8A). Similar results were obtained with the construct pMIR-Report-3′-UTR-S2 containing seed region 2 (SDR2) of *MCPH1* in a sense orientation and the pcDNA3/pre-miR-27a/*EGFP* construct (Figure 8B), corroborating the above observations that both seed regions bind to miR-27a. To further validate the interaction of miR-27a with SDR1 or SDR2, we mutated each of the two SDRs by site-directed mutagenesis (Table S5 in File S1). We then co-transfected the pMIR-Report-3′-UTR-M1 construct containing the mutated SDR1 sequence or pMIR-Report-3′-UTR-M2 construct containing the mutated SDR2 sequence separately in KB cells along with pcDNA3/pre-miR-27a/*EGFP*, and the luciferase activity was measured after 2 days. As compared to controls, the luciferase activity did not change in cells transfected with either pMIR-Report-3′-UTR-M1 and pcDNA3/pre-miR-27a/*EGFP* or pMIR-Report-3′-UTR-M2 and pcDNA3/pre-miR-27a/*EGFP*, suggesting that both the seed regions in the 3′-UTR of *MCPH1* are important for the interaction with miR-27a (Figure 8).

**Figure 9:**
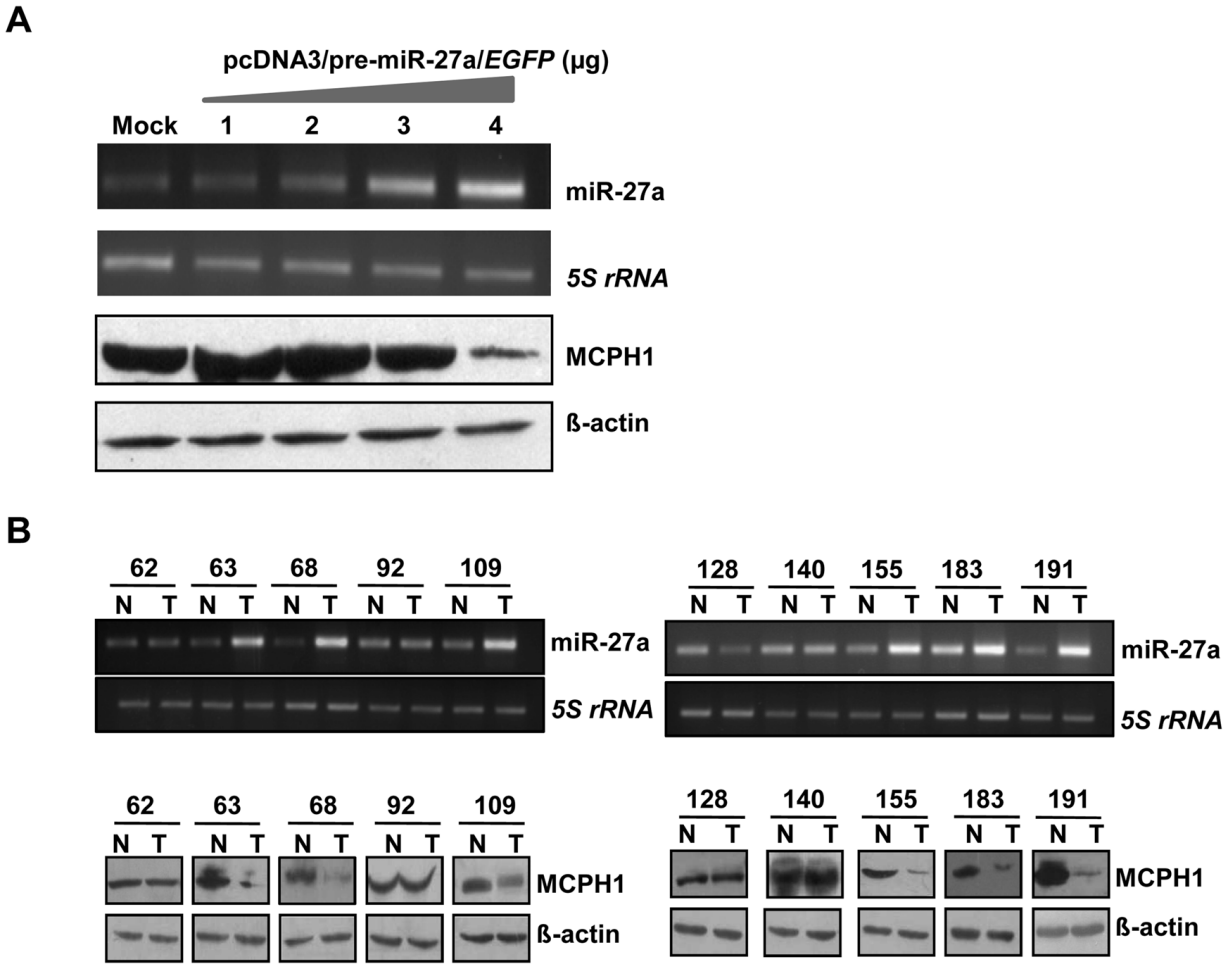
miR-27a negatively regulates MCPH1. (A) miR-27a negatively regulates MCPH1 level in KB cells. Representative images of the correlative expression of MCPH1 protein level after the transient transfection of the pcDNA3/pre-miR-27a/*EGFP* construct in KB cells. Note the reduced expression of MCPH1 upon overexpression (4 µg) of pcDNA3/pre-miR-27a/*EGFP*. Mock lane represents transfection with the empty vector pcDNA3/*EGFP*. *5S rRNA* and ß–actin were loading controls for RT-PCR and Western blotting respectively. (B) The correlative expression analysis of miR-27a and MCPH1 in 10 paired OSCC samples. Note a negative correlation between the levels of miR-27a and MCPH1 in six matched samples: 63, 68, 109, 155, 183 and 191. However, no correlation between the levels of miR-27a and MCPH1 was observed in three matched samples: 62, 92 and 140. In the remaining one matched sample (pt# 128), the level of miR-27a was downregulated in tumor, but the level of MCPH1 was unchanged in the tumor tissue. *5S rRNA* and ß–actin were loading controls for RT-PCR and Western blotting respectively. Abbreviations: N, normal oral tissue; and, T, tumor oral tissue. The numbers refer to patient numbers.

Further to see the effect of miR-27a on the level of endogenous MCPH1, we transiently transfected the pcDNA3/pre-miR-27a/*EGFP* or pcDNA3/*EGFP* (empty vector) separately in KB cells and after 2 days the cells were lysed and the level of MCPH1 was determined by Western blot analysis. Although no dose-dependent relationship between the quantity of transfected pcDNA3/pre-miR-27a/*EGFP* construct and the level of MCPH1 was found, a reduced level of MCPH1 was observed when the cells were transiently transfected with 4 µg of the pcDNA3/pre-miR-27a/*EGFP* construct (Figure 9A), suggesting that the miR-27a indeed negatively regulates MCPH1 level *in vitro*.

We then wanted to see if a negative correlation exists between endogenous levels of miR-27a and MCPH1 in OSCC samples. We selected a panel of 10 matched normal oral and OSCC tissues and determined the levels of miR-27a and MCPH1 using semi-quantitative RT-PCR and Western blotting, respectively. A negative correlation between the levels of miR-27a and MCPH1 was found in six matched samples: pt# 63, 68, 109, 155, 183 and 191 (Figure 9B). However, no correlation between the levels of miR-27a and MCPH1 was observed in three pairs: pt# 62, 92 and 140. In the remaining pair (pt# 128), the level of miR-27a was downregulated in tumor, but the level of MCPH1 remained unchanged between normal and tumor tissues (Figure 9B).

### No Correlation in the Levels of *MCPH1* and *BRCA1*

As stated above, the knockdown of MCPH1 in HEK293 cells lowers the transcript level of *BRCA1*. In order to examine if such a correlation also exists in OSCC, we determined their expression levels in 24 matched OSCC samples by quantitative real-time RT-PCR and the correlation was studied by Pearson’s correlation analysis. The analysis showed no inverse or positive correlation between the levels of *BRCA1* and *MCPH1* transcripts, suggesting that *BRCA1* is not regulated by MCPH1 in OSCC (Figure S17 in File S2).

### No Correlation of *MCPH1* Expression Level with Clinico-pathological Parameters

It is important to note that tumor suppressors can serve as potential biomarkers with prognostic values. Hence, we analyzed the correlation of both the transcript and protein levels of MCPH1 with clinico-pathological parameters such as gender of the patients, T stages of the tumors, age of the patients and site of the tumors by Fischer’s exact test. No statistical correlation was observed between the transcript or protein level of MCPH1 and any of the four clinico-pathological parameters (Figures S18 and S19 in File S2).

## Discussion

Genomic aberrations at the MCPH1 locus have been reported previously in cancers [4], [14], [15]. Using a high-density array genomic hybridization (aCGH) technique, Rai et al. [4] have previously reported a high frequency of decreased *MCPH1* DNA copy numbers in 35/87 (40.23%) advanced epithelial ovarian cancer samples. Based on its dual role in TERT (telomerase reverse transcriptase) repression and cell cycle checkpoint regulation, downregulation at the transcript and protein levels in breast, prostate and ovarian cancers as well as the presence of a homozygous mutation in 1/10 breast tumor samples, Rai et al. [4] have previously suggested that *MCPH1* may function as a tumor suppressor. In the present study, we have carried out for the first time a comprehensive analysis to test if the *MCPH1* gene functions as a tumor suppressor in OSCC, using a total of 91 OSCC samples, 2 epithelial dysplasia cases and 5 cancer cell lines. The results showed that MCPH1 exhibits three hallmarks of tumor suppressors [32], such as the presence of LOH, somatic mutations and promoter methylation in tumor samples, although at a lower rate. In addition, as expected for TS genes, *MCPH1* overexpression reduced cell proliferation, colony formation in soft agar assay and tumor growth in nude mice, suggesting that it indeed functions as a tumor suppressor gene, in addition to its role in brain development.

Mutations in the *MCPH1* gene have been observed previously in MCPH, PCC syndrome and a few cancers. Thirteen homozygous mutations have been reported in MCPH patients [2], [33]–[37]. A homozygous mutation c.427_428insA(p.Thr143fsX5) in *MCPH1* causes PCC [3]. A 38 bp deletion in exon 10 was observed in 1/10 breast cancer samples [4]. Two heterozygous mutations c.321delA(p.Lys107fsX39) and c.1402delA(p.Thr468fsX32) reported in OSCC cell lines in the present study have also been reported in endometrial cancer in a heterozygous state [16]. A novel homozygous nonsense mutation c.1561G>T(p.Glu521X) was observed in an OSCC sample in the present study. Further, with a novel mutation reported here, the total number of mutations in the *MCPH1* gene is 19 (Table S7 in File S1). It is interesting to note that except for c.321delA(p.Lys107fsX39) and c.1402delA(p.Thr468fsX32), none are recurrent mutations.

Hypermethylation of CpG islands located in promoter regions of TS genes is now firmly established as an important mechanism for their inactivation [38]. For example, TS genes involved in DNA repair such as *BRCA1*, *MLH1* and *MGMT* show promoter hypermethylation in human cancers [38]. Interestingly, MCPH1, also involved in DNA repair, showed promoter methylation, albeit in a low number of 4/40 OSSC samples (viz., pt# 80, 116, 177 and 202), and three of these samples (viz., pt# 116, 177 and 202) also showed downregulation of its transcript (Table S8 in File S1). The fourth OSCC sample (pt# 80) showed promoter methylation of CpGII, but there was no change in the transcript levels of *MCPH1* between the matched normal and tumor samples (Table S8 in File S1). We believe that this discrepancy could be due to the occurrence of intra-tumor heterogeneity. It is well known that human tumors often display startling intra-tumor heterogeneity in various features including histology, gene expression, genotype, metastatic and proliferative potential [39].

To the best of our knowledge, this is the first report of methylation of CpG islands of the *MCPH1* promoter in any cancer. A low rate of promoter methylation does not seem to be unique to *MCPH1* only as low (<10%) methylation of TS genes have been reported before. For example, a low rate of promoter methylation was observed for the *TUSC3* (9/105 samples), *MGMT* (8/105 samples) and *CDKN2B* (2/105 samples) genes in breast tumors [40]. Moreover, while our manuscript was in preparation, Shi and Su [41] reported the characterization of the *MCPH1* promoter (GenBank accession# JN573214.1) in HeLa and HEK293 cells by luciferase assay. Their promoter was ∼1.96 kb large in contrast to our *in silico* identified *MCPH1* promoter (ID: 39666) of ∼1 kb. When we performed the sequence alignment of both the promoter sequences using the ClustalW2 alignment program (http://www.ebi.ac.uk/Tools/msa/clustalw2/), we found that out of 1 kb of the *MCPH1* promoter (ID: 39666), 0.862 kb was identical to that of JN573214.1. Additionally, we also analyzed the promoter JN573214.1 using the CpG plot program and found it to harbor the same two CpG islands which were accounted for promoter methylation in our study. Hence, our study has accounted methylation of all the CpG islands.

We have shown that the tumor suppressive function of MCPH1 in KB cells is due to induction of apoptosis as characterized by elevated CASP3 activation, which is an important event in apoptosis [42]. Consistent with our results, it has been shown that the knockdown of MCPH1 decreases the level of CASP3 in HEK293 cells [7]. Yang et al. [7] have further shown that the knockdown of MCPH1 in U2OS cells decreases apoptosis as seen by decreased annexin staining, suggesting that MCPH1 induces apoptosis. Since we did not correlate the expression levels of MCPH1 and CASP3 in OSCC samples, we performed a literature search to find if CASP3 expression has been reported in oral cancer. We found that CASP3 protein has been shown to be downregulated in 79/87 (90.80%) of tongue squamous cell carcinoma [43]. In contrary to this study, overexpression of CASP3 (protein) has been reported in a study using 39 samples of normal oral epithelium and 54 oral squamous cell carcinomas [42]. These two conflicting results lead us to conclude that in some cases a higher CASP3 expression could be tolerated in oral cancer by evading apoptosis through some unknown mechanism. In addition to apoptosis, MCPH1 overexpression reduced the anchorage-independent growth in soft agar and cell invasion of KB cells. Hence, our results showed for the first time that the MCPH1 overexpression brings anoikis (cell detachment induced apoptosis seen as reduced growth in soft agar). Further, MCPH1 is a DNA repair protein and hence, it would be interesting to examine the functional role of MCPH1 in the overexpressing stable clones after an external stimulation of DNA damage. Though our work has not addressed this functional aspect of MCPH1 in the generated stable clones, our results are consistent with observations of Yang et al. [7] that MCPH1 possesses apoptotic functions under physiological conditions. To our knowledge, no functional analysis has been previously performed to validate the tumor suppressor function of MCPH1. We chose KB cells (a cancer cell line) to validate this tumor suppressor function in a general context of cancer. KB cells are HeLa derivatives and are not a specific model for oral squamous cell carcinoma [44, http://www.atcc.org/CulturesandProducts/CellBiology/MisidentifiedCellLines/tabid/683/Default.aspx]. Two OSCC cell lines (SCC084 and SCC131) were resistant to G418 selection and therefore were not used to generate MCPH1 overexpressing stable clones. The studies conducted in KB cells demonstrate that MCPH1 possesses tumor suppressive properties due to its ability to reduce growth in vitro and in vivo. Both HeLa and oral cancer cell lines are epithelial, meaning that overexpression studies conducted in KB cells may mimic the changes that MCPH1 would cause in oral epithelial cells.

Studies have shown the involvement of microRNAs in post-transcriptional regulation of genes in cancer [45]. We have shown for the first time that *MCPH1,* being a tumor suppressor gene, is regulated by miR-27a in oral cancer and uses both of its target seed regions in its 3′-UTR. Intriguingly, we observed that the downregulation of MCPH1 by miR-27a occurred only when the KB cells were transfected with 4 µg of the pcDNA3/pre-miR-27a/*EGFP* construct. The lower concentration of the construct failed to downregulate MCPH1 (Figure 9A). This is due to the fact that miR-27a could have other targets and hence, pre-miRNA processed at the 4 µg concentration was optimal for MCPH1 downregulation. Moreover, our results suggest that miR-27a is oncogenic in the context of MCPH1. Few studies have already reported the oncogenic role of miR-27a. For example, it has been shown to be upregulated in 30/51 HNSCC tissues and 3 HNSCC cell lines such as FaDu, UTSCC-8 and UTSCC-42a [46]. Further, it inhibits *SPRY2* mRNA, a crucial inhibitor of the Ras/MAPK signaling pathway, and promotes cell proliferation and migration in pancreatic cancer cell lines such as PANC-1 and MIA PaCa-2 [47].

Centrosome amplification leads to the formation of more than two spindle poles [48]. The multipolar spindles thus formed result in an increased frequency of chromosome segregation errors (chromosome instability) which promote tumorigenesis [48]. Further, centrosome amplification is seen in cancers of the breast, prostate, head and neck, ovary and cervix [48]. Hence, genes involved in the maintenance of centrosomal number have a crucial role in cancer. It is interesting to note that all the eight primary microcephaly (MCPH) genes such as *MCPH1*, *WDR62* (WD repeat domain 62), *CDK5RAP2* (cyclin-dependent kinase 5 regulatory associated protein 2), *CEP152* (centrosomal protein 152 kDa), *ASPM* (abnormal spindle-like, microcephaly-associated protein), *CENPJ* (centromeric protein J), *STIL* (SCL/TAL1-interrupting locus) and CEP135 (centrosomal protein 135 kDa) are centrosomal proteins [49]–[52]. Centrosome amplification has been reported in fibroblast cells from human patients harboring *CEP152* or *CEP135* mutations [52], [53]. Furthermore, depletion of MCPH1 in U2OS cells leads to ionizing radiation induced centrosomal amplification [8]. MCPH1 negatively regulates AURKA which is known to induce centrosomal amplification [8]. It is therefore not surprising to note that MCPH1, required for the maintenance of centrosome number, has a role in development of cancers.

It is also interesting to note that, in addition to MCPH1, few other MCPH genes have shown relevance in human cancers. For example, the MCPH5 gene *ASPM* is highly expressed in 175 gliomas as compared to three normal brain tissues [54]. A higher expression of *ASPM* was also seen in 4/7 recurrent gliomas compared to their corresponding primary gliomas [54]. In addition to *ASPM*, the MCPH7 gene *STIL* has also been found to be associated with human cancer. A chromosomal translocation involving the *STIL* gene is reported in T-cell acute lymphoblastic leukemia (T-ALL) [50]. The translocation causes a 100 kb deletion at 1p32, leading to the fusion of exon 3 of *TAL-1* with exon 1 of *STIL*, thus activating the *TAL-1* expression. Furthermore, the overexpression of *STIL* is associated with metastasis of adenocarcinomas of the lung, breast, ovary, prostate and colon [50].

Our study has shown that the overexpression of MCPH1 exhibits anti-tumorigenic effects, and therefore it is alluring to propose that the restoration of MCPH1 could be a therapeutic strategy to treat OSCC. Although farfetched as of now, the *MCPH1* gene can be delivered through viral vectors by intra-tumoral injections and topical applications as gene therapy mechanisms [55]. The design of anti-mir oligos for miR-27a, which can upregulate *MCPH1*, can also be used for therapy. However, the precision of these assumptions require further in depth and extensive pre-clinical validations.

In summary, the results of the present study have suggested that the primary microcephaly gene *MCPH1* shows several hallmarks of TS genes and functions as a tumor suppressor in OSCC, in addition to its role in brain development. We have for the first time shown that miR-27a targets *MCPH1* and regulates its level. It is interesting to note that none of the other eight MCPH genes have been shown to be regulated by miRNAs yet. Our study will be useful in designing novel therapeutic methods for the treatment of OSCC.

## Supporting Information

File S1 **Supporting multiple Tables.**
(DOCX)

File S2 **Supporting multiple figures.**
(DOCX)
