# Supplementary material for: Primary Microcephaly Gene MCPH1 Shows Signatures of Tumor Suppressors and Is Regulated by miR-27a in Oral Squamous Cell Carcinoma
Source: PLoS One. 2013 Mar 5;8(3):e54643. doi: 10.1371/journal.pone.0054643 (PMC3589425; doi:10.1371/journal.pone.0054643)
Supplement: File S1 — Supporting multiple Tables. (DOCX) [file pone.0054643.s001.docx]

**File S1:** Supporting multiple Tables

**Primary microcephaly gene *MCPH1* shows signatures of tumor suppressors and is regulated by miR-27a in oral squamous cell carcinoma**

Thejaswini Venkatesh^1^, Mathighatta Nagaraj Nagashri^1^, Shivananda S. Swamy^2^, S.M. Azeem Mohiyuddin^3^, Kodaganur S. Gopinath^2^ and Arun Kumar^1^

^1^Department of Molecular Reproduction, Development and Genetics, Indian Institute of Science, Bangalore 560012, Karnataka, India. ^2^Department of Surgical Oncology, Bangalore Institute of Oncology, Bangalore 560027, Karnataka, India. ^3^Department of Otolaryngology and Head and Neck Surgery, R.L. Jalappa Hospital and Research Centre, Kolar 563101, Karnataka, India.

**Corresponding Author**

Prof. Arun Kumar

Department of Molecular Reproduction, Development and Genetics

Indian Institute of Science

Bangalore 560012

India

Email: karun@mrdg.iisc.ernet.in

Telephone: 91-80-2293 2998

Fax: 91-80-2360 0999

Table S1: A summary of clinico-pathological features of patients included in the study.

| **Characteristics** | **No. of patients (n=93)** |
| --- | --- |
| **Median age/range**  ≤ 54  ≥ 55  **Gender**  Males  Females  **Site of cancer**  Buccal mucosa  Tongue-anterior 2/3  Others^a^  **Tumor classification**  T1  T2  T3  T4  Epithelial Dysplasia  **Lymph node status**  positive  negative  **Differentiation**  Well  Moderate  Poor  **Tobacco usage**  Tobacco positive#  Tobacco negative  **Treatment**  Surgery  Radiotherapy  Chemotherapy | 54 yrs/32-80  47 (50.54%)  46 (49.46%)  36 (38.71%)  57 (61.29%)  57 (61.29%)  15 (16.12%)  19 (20.43%)  8 (8.60%)  25 (26.88%)  22 (23.65%)  36 (38.70%)  2 (2.58%)  72 (79.12%)  19 (20.88%)  43 (47.25%)  35 (38.46%)  13 (14.28%)  85 (91.39%)  8 (8.60%)  93 (100%)  87 (93.54%)  3 (3.22%) |

^a^ The sites of cancer include lower alveolus, floor of the mouth, lip and

retromolar trigone. # indicates addiction to tobacco, bidi or cigarettes for at least

5 years.

**Table S2**: Details of clinico-pathological features of 93 OSCC and ED patients included in the study.

| **Pt #** | **Age/Sex** | **Site of tumor** | **TNM** | **Differentiation** | **Tobacco usage** | **Treatment** |
| --- | --- | --- | --- | --- | --- | --- |
| 5 | 41M | SCC BM | T3N1M0 | Moderate | Chronic Tobacco | Surgery+ post operative RT |
| 8 | 50F | SCC BM | T3N1M0 | Well | Chronic Tobacco | Surgery+ post operative RT |
| 9 | 45M | Tongue-  anterior 2/3 | T4N2M0 | Well | Chronic Tobacco | Neoadjuvant CT+ surgery |
| 11 | 60F | SCC BM | T4N1MX | Moderate | Chronic Tobacco | Surgery+ post operative RT |
| 14 | 60F | SCC BM | T4N1MX | Moderate | Chronic Tobacco | Surgery+ post operative RT |
| 13 | 65F | SCC BM | T4N0M0 | Well | Chronic Tobacco | Surgery+ post operative RT |
| 12 | 60F | SCC BM | T4N1MX | Moderate | Chronic Tobacco | Surgery+ post operative RT |
| 15 | 55F | SCC BM | T3N1M0 | Well | Chronic Tobacco | Surgery+ post operative RT |
| 17 | 55F | SCC BM | T4N1MX | Well | Chronic Tobacco | Surgery+ post  operative RT |
| 18 | 60F | SCC BM | T3N2M0 | Well | Chronic Tobacco | Surgery+ post operative RT |
| 19 | 48F | SCC BM | T4N2bM0 | Well | Chronic Tobacco | Surgery+ post operative RT |
| 20 | 50F | Lower alveolus | T4N1M0 | Moderate | Chronic Tobacco | Surgery+ post operative RT |
| 21 | 32F | SCC BM | T4N1M0 | Moderate | Chronic Tobacco | Surgery+ post operative RT |
| 22 | 60F | SCC BM | T2N1M0 | Well | Chronic Tobacco | Surgery+ post operative RT |
| 26 | 45M | Lower alveolus | T3N1M0 | Well | Chronic Tobacco | Surgery+ post operative RT |
| 27 | 40F | SCC BM | T4N1M0 | Well | Chronic Tobacco | Surgery+ post operative RT |
| 30 | 65F | SCC BM | T4N1M0 | Moderate | Chronic Tobacco | Surgery+ post operative RT |
| 34 | 65F | SCC BM | T3N1M0 | Moderate | Chronic Tobacco | Surgery+ post operative RT |
| 38 | 40M | RMT | T4N1M0 | Moderate | Chronic Tobacco | Surgery+ post operative RT |
| 39 | 55F | Lower alveolus | T4N1M0 | Well | Chronic Tobacco | Surgery+ post operative RT |
| 40 | 50F | SCC BM | T2N1M0 | Well | Chronic Tobacco | Surgery+ post operative RT |
| 41 | 57F | RMT | T1N0M0 | Poor | Chronic Tobacco | Surgery+ post operative RT |
| 44 | 60F | SCC BM | T3N1M0 | Well | Chronic Tobacco | Surgery+ post operative RT |
| 47 | 45F | SCC BM | T2N1M0 | Well | Chronic Tobacco | Surgery+ post operative RT |
| 50 | 50M | SCC BM | T4N2bM0 | Well | Chronic Tobacco | Surgery+ post operative RT |
| 52 | 70M | ED | N/A | N/A | No Tobacco | Surgery |
| 53 | 38F | SCC BM | T2N1M0 | Well | Chronic Tobacco | Surgery+ post operative RT |
| 54 | 40M | Tongue-anterior 2/3 | T4N1M0 | Well | Chronic Tobacco | Surgery |
| 55 | 40F | SCC BM | T4N1M0 | Well | Chronic Tobacco | Surgery+ post operative RT |
| 56 | 50F | ED | N/A | N/A | No Tobacco | Surgery |
| 57 | 62F | SCC BM | T1N0M0 | Well | Chronic Tobacco | Surgery+ post operative RT |
| 59 | 70F | RMT | T4N2bM0 | Well | Chronic Tobaco | Surgery+ post operative RT |
| 60 | 40F | SCC BM | T4aN1MX | Poor | Chronic Tobacco | Surgery+ post operative RT |
| 62 | 35F | SCC BM | T3N0M0 | Well | Chronic Tobacco | Surgery+ post operative RT |
| 63 | 40F | SCC BM | T4aN1MX | Moderate | Chronic Tobacco | Surgery+ post operative RT |
| 65 | 55F | SCC BM | T2N1MX | Well | Chronic Tobacco | Surgery+ post operative RT |
| 66 | 55F | SCC BM | T3N2bM0 | Well | Chronic Tobacco | Surgery+ post operative RT |
| 67 | 38F | SCC BM | T2N1M0 | Well | Chronic Tobacco | Surgery+ post operative RT |
| 68 | 65M | Lower alveolus | T3N2bM0 | Well | Chronic Tobacco | Surgery+ post operative RT |
| 69 | 70F | SCC BM | T2N1M0 | Moderate | Chronic Tobacco | Surgery+ post operative RT |
| 70 | 40F | SCC BM | T2N1M0 | Well | Chronic Tobacco | Surgery+ post operative RT |
| 71 | 51M | SCC BM | T4N1M0 | Well | Chronic Tobacco | Surgery+ post operative RT |
| 72 | 40F | SCC BM | T4aN2bM0 | Well | Chronic Tobacco | Surgery+ post operative RT |
| 73 | 50F | SCC BM | T3N1M0 | Well | Chronic Tobacco | Surgery+ post operative RT |
| 74 | 54F | SCC BM | T3N0M0 | Well | No Tobacco | Neoadjuvant CT+surgery |
| 75 | 62M | Tongue-anterior 2/3 | T4N2M0 | Poor | Chronic Tobacco | Surgery+ post operative RT |
| 76 | 47M | Tongue-anterior 2/3 | T4N2M0 | Moderate | Chronic Tobacco | Neoadjuvant CT+surgery |
| 77 | 40F | SCC BM | T4aN1MX | Well | Chronic Tobacco | Surgery+ post operative RT |
| 79 | 67M | SCC BM | T3N1MX | Well | Chronic Tobacco | Surgery+ post operative RT |
| 80 | 67M | FOM | T4N0M0 | Well | Chronic Tobacco | Surgery+ post operative RT |
| 83 | 50M | Lower alveolus | T3N2bMX | Moderate | Chronic Tobacco | Surgery+ post operative RT |
| 92 | 50F | SCC BM | T4aN2bM0 | Well | Chronic Tobacco | Surgery+ post operative RT |
| 95 | 52M | FOM | T3N2bM0 | Moderate | Chronic Tobacco | Surgery+ post operative RT |
| 101 | 48F | SCC BM | T2N1MX | Moderate | Chronic Tobacco | Surgery+ post operative RT |
| 108 | 56M | Upper alveolus | T4N2bM0 | Poor | Chronic Tobacco | Surgery+ post operative RT |
| 109 | 45M | SCC BM | T3N1M0 | Moderate | Chronic Tobacco | Surgery+ post operative RT |
| 110 | 45M | SCC BM | T2N1M0 | Moderate | Chronic Tobacco | Surgery+ post operative RT |
| 113 | 80F | SCC BM | T1N0M0 | Well | Chronic Tobacco | Surgery+ post operative RT |
| 114 | 68F | SCC BM | T3N1M0 | Moderate | Chronic Tobacco | Surgery+ post operative RT |
| 115 | 55F | SCC BM | T4N1M0 | Moderate | Chronic Tobacco | Surgery+ post operative RT |
| 116 | 40M | RMT | T4N0M0 | Poor | Chronic Tobacco | Surgery+ post operative RT |
| 121 | 70F | Lower alveolus | T4N1M0 | Poor | Chronic Tobacco | Surgery+ post operative RT |
| 125 | 57M | Tongue-anterior 2/3 | T2N1M0 | Moderate | No Tobacco | Surgery+ post operative RT |
| 127 | 75F | Lip | T1N0M0 | Well | Chronic Tobacco | Surgery+ post operative RT |
| 128 | 60F | SCC BM | T2N0M0 | Moderate | Chronic Tobacco | Surgery+ post operative RT |
| 133 | 71F | SCC BM | T2N0M0 | Moderate | Chronic Tobacco | Surgery+ post operative RT |
| 135 | 41M | SCC BM | T2N0M0 | Moderate | Chronic Tobacco | Surgery+ post operative RT |
| 139 | 71F | SCC BM | T4N1M0 | Moderate | Chronic Tobacco | Surgery+ post operative RT |
| 140 | 70M | Lip | T4N1M0 | Well | Chronic Tobacco | Surgery+ post operative RT |
| 143 | 43M | SCC BM | T3N1M0 | Moderate | Chronic Tobacco | Surgery+ post operative RT |
| 144 | 39M | SCC BM | T2N1M0 | Poor | Chronic Tobacco | Surgery+ post operative RT |
| 155 | 60F | SCC BM | T3N0MX | Moderate | Chronic Tobacco | Surgery+ post operative RT |
| 156 | 75M | Tongue-anterior 2/3 | T2N0M0 | Moderate | Chronic Tobacco | Surgery+ post operative RT |
| 158 | 50F | SCC BM | T2N1M0 | Moderate | Chronic Tobacco | Surgery+ post operative RT |
| 157 | 42M | Tongue-anterior 2/3 | T1N0M0 | Well | Chronic Tobacco | Surgery+ post operative RT |
| 159 | 40F | Lower alveolus | T4N0M0 | Moderate | Chronic Tobacco | Surgery+ post operative RT |
| 165 | 65M | Tongue-anterior 2/3 | T2N1M0 | Well | Chronic Tobacco | Surgery+ post operative RT |
| 166 | 50F | SCC BM | T2N1M0 | Poor | Chronic Tobacco | Surgery+ post operative RT |
| 174 | 70F | SCC BM | T2N0M0 | Well | Chronic Tobacco | Surgery+ post operative RT |
| 175 | 72M | SCC BM | T4N1M0 | Moderate | Chronic Tobacco | Surgery+ post operative RT |
| 177 | 59M | FOM | T2N1M0 | Poor | Chronic Tobacco | Surgery+ post operative RT |
| 180 | 71M | Tongue-anterior 2/3 | T3N1M0 | Poor | Chronic Tobacco | Surgery+ post operative RT |
| 181 | 45M | Tongue-anterior 2/3 | T2N1M0 | Moderate | Chronic Tobacco | Surgery+ post operative RT |
| 182 | 65M | Tongue-anterior 2/3 | T1N0M0 | Well | No Tobacco | Surgery+ post operative RT |
| 183 | 50M | Tongue-anterior 2/3 | T1N1M0 | Moderate | Chronic Tobacco | Surgery+ post operative RT |
| 191 | 45F | Lower alveolus | T2N0M0 | Moderate | Chronic Tobacco | Surgery+ post operative RT |
| 192 | 56M | Tongue-anterior 2/3 | T1N1M0 | Moderate | No Tobacco | Surgery+ post operative RT |
| 194 | 48F | Lower alveolus | T4N2bM0 | Well | Chronic Tobacco | Surgery+ post operative RT |
| 202 | 37F | Tongue-anterior 2/3 | T2N2M0 | Moderate | No Tobacco | Surgery+ post operative RT |
| 206 | 44M | Tongue-anterior 2/3 | T3N2bM0 | Poor | Chronic Tobacco | Surgery+ post operative RT |
| 212 | 65F | SCC BM | T4N1M0 | Poor | Chronic Tobacco | Surgery+ post operative RT |
| 214 | 60F | SCC BM | T2N2M0 | Well | Chronic Tobacco | Surgery+ post operative RT |
| 219 | 68M | SCC BM | T3N1M0 | Poor | No Tobacco | Surgery+ post operative RT |

Abbreviations: Pt#, patient number; M, male; F, female; ED, epithelial dysplasia; BM, buccal mucosa; SCC BM, squamous cell carcinoma of the buccal mucosa; RMT, retromolar trigone; FOM, floor of the mouth; RT, radiotherapy; and, CT, chemotherapy.

Table S3: Primers and their PCR conditions for the mutation analysis of the *MCPH1* gene*.*

| **Exon #** | **Sequence (5´ to 3´)** | **Amplicon size (bp)** | **Annealing temperature (^o^C)** |
| --- | --- | --- | --- |
| 1^a^ | F: CACCTACAGAGAAATCCCGGAAAC  R:AGGGGTGCCGGTCCTCAAACTC | 258 | 60 |
| 2 | F:CTGTGCCGGCCTCGGTTTAC  R:CTCCCACCGCTTACCCATTG | 324 | 64 |
| 3 | F:CTTGTGTGTATGATTCACCGTTG  R:CAAATTACTAGGTGAGGTAAGAG | 442 | 60 |
| 4 | F:GCTAATACATGTGCAGATTTAGTGC  R:TCCCATGGCCAACCACAGGCT | 404 | 64 |
| 5 | F:CCTGCCTTAAGCAGTTGCAGTAC  R:ATCAGCTCTCTCATGCTGAACCC | 265 | 64 |
| 6 | F:TGAAGTATGAAGGCACTTTTTGGTC  R:GAAAGCTTTCCACCATAATTGAATCG | 262 | 64 |
| 7 | F:TAGAATCACCTATGATTAATAGGAGGAC  R:CACATCAGAAGTTGCTACATGAAATTC | 281 | 64 |
| 8 | F:GAGAAGAACTCAAGTGTGGTTAATAG  R:CAAACGATACTTCTCTTCAAACGTC | 464 | 64 |
| 8^a^ | F:TGCAGGTAAAGTAGTCACCCCTG  R:CTTCTTAGAAAAGACTTCTGCAGCTC | 433 | 55 |
| 8 | F:CTGGAGGCTCTTAGCTGTGG  R:CATCATGTCCTTTTGGAAGAGC | 415 | 64 |
| 8 | F:GAAGCCCTAAGGTGTTGTAGAC  R:AGGTGACTTGGAAAAGGAGATTC | 342 | 64 |
| 9 | F:GACGGAGTTGATGCTGTAGAAC  R:GGTTTATGTTTCATTGACCCACAG | 435 | 64 |
| 10 | F:GCTGGCTAACTGGTGGAACAG  R:CCTAAAGGCACCCAGAATTAGAG | 400 | 64 |
| 11 | F:GTGTAACTGCTTTGATGGGCATG  R:TGCAGATCCACAAGGGTGCACG | 345 | 64 |
| 12 | F:CTTGGTTTATTGCCTGCTAAGG  R:CTGCATTTACCATCGTAAAACAAC | 177 | 64 |
| 13 | F:ACGCTATGGACTGGAGTGGTCC  R:CAGATCTGGACCACACCACAGCG | 340 | 64 |
| 14 | F:AGGTATGTGTGCTCTATGGACGTGG  R:GTCACCACAGGCCAGTGAGGTCAC | 238 | 59 |

Abbreviations: F, forward primer; R, reverse primer; and, bp, base pair. ^a^work with 5%

DMSO. A typical PCR consists of 1X buffer, 1.5 mM of MgCl_2_, 0.2 nM of each dNTP, 50 ng of each

primer, 1 U of *Taq* DNA polymerase (Sigma-Aldrich, St Louis, MO) and 50 ng of genomic DNA.

Table S4: Primers and their PCR conditions used in RT-PCR and COBRA.

| **Name of amplicon** | **Sequence (5´ to 3´)** | **Amplicon size (bp)** | **Annealing temperature**  **(^o^ C)** |
| --- | --- | --- | --- |
| *GAPDH* | F:GAAGGGTGAAGGTCGGAGTC  R:GAAGATGGTGATGGGATTTC | 226 | 60 |
| *MCPH1* | F:TCACCACAGCGCAATGGAGAAGAGA  R:ATCACGTGAAATGTTCAAAGGTGCTTC | 145 | 62 |
| *ß-actin* | F:GAGCCTCGCCTTTGCCGATCC  R:GACCCATGCCCACCATCACGC | 196 | 70 |
| miR-27a | RT6-miR- 27a:TGTCAGGCAACCGTATTCACCGTGAGTGGTGCGGAA  MP-fw  Short-miR-27a: CGTCAGATGTCCGAGTAGAGGGGGAACGGCGTTCACAGTGGCTAAG  MP-rev  MP-fw: TGTCAGGCAACCGTATTCACC  MP-rev: CGTCAGATGTCCGAGTAGAGG | 81 | 58 |
| *5S rRNA* | F:GCCCGATCTCGTCTGATCT  R:AGCCTACAGCACCCGGTATT | 94 | 60 |
| *BRCA1* | F:ATCAGATTCAGGGTCATCAGAGAAG  R:CACAGTTGCTCTGGGAGTCTTCAG | 178 | 64 |
| First PCR of CpGI^a^ | F:TTTGAGGTTTGGAGGTATTTTTG R:TAAATTTTTCTCTTCTCCTAAAAAT | 453 | 54 |
| Second PCR of CpGI^a^ | F:GGGTTATTTTGTGGGGGTTTGAAG R:CCCCTAACCCCTAAATTAACCTTC | 373 | 60 |
| First PCR of CpGII^a^ | F:TTTTAGTTTAGGTGAGTTTAGATGAGG R:AACAAAACTCCTCCCACAAAATCCC | 465 | 54 |
| Second PCR of CpGII^a^ | F:AAATAGAAGGTTAATTTAGGGGTTAGG R:AAAACAAACAACAAAAAATACCTCACC | 337 | 54 |
| *ASPM* (exon 1)^b^ | F:AGGAGATCCAGGAGGGGTCTCG  R:GCCTGGAGCACGCTCCTCCTG | 356 | 60 |

Abbreviations: F, forward primer; R, reverse primer; bp, base pair. ^a^indicates PCR works with 2 mM of MgCl_2_; the remaining PCRs work with 1.5 mM MgCl_2_. ^b^ indicates PCR works with 5% DMSO.

Table S5: Details of constructs used in the present study.

| **Construct** | **Primer sequence (5’ to 3’)** | **Amplicon size (bp)** | **Annealing temperature (^o^ C)** |
| --- | --- | --- | --- |
| pcDNA3/  pre-miR-27a/*EGFP* | F:CCGAAAGCTTCCTGGGGATGGGATTTGCTTC  *Hind* III  R: TCGACTCGAGACCCCTGTTCCTGCTGAACTG  *Xho I* | 366 | 64 |
| pMIR-Report-3’-UTR-S | F:GATAGAGCTCGCGGAGGCCGGGTCAGCCAAG  *Sac* I  R:GACAAAGCTTGAAATGAGACAGTTAGTTTTTATTTATTCC  *Hind* III | 5456 | 55 |
| pMIR-Report-3’-UTR-AS | F: GACAAAGCTTGCGGAGGCCGGGTCAGCCAAG  *Hind* III  R: GATAGAGCTCGAAATGAGACAGTTAGTTTTTATTTATTCC  *Sac* I | 5456 | 55 |
| pMIR-Report-3’-UTR-M1F | F:CAAATGAGAAACAAAAC**CACA**AAGAGAAGGAACTGG  R:CCAGTTCCTTCTCTT**TGTG**GTTTTGTTTCTCATTTG | 5456 | 55 |
| pMIR-Report-3’-UTR-M2F | F:GAATCTGGGCAGCAC**CACA**AAACCAACATTTCTTCAG  R:CTGAAGAAATGTTGGTTT**TGTG**GTGCTGCCCAGATTC | 5456 | 55 |
| pMIR-Report-3’-UTR-MF | F:CAAATGAGAAACAAAAC**CACA**AAGAGAAGGAACTGG  R:CCAGTTCCTTCTCTT**TGTG**GTTTTGTTTCTCATTTG  F:GAATCTGGGCAGCAC**CACA**AAACCAACATTTCTTCAG  R:CTGAAGAAATGTTGGTTT**TGTG**GTGCTGCCCAGATTC | 5456 | 55 |
| pMIR-Report-3’-UTR-S1 | F:GATAGAGCTCGCGGAGGCCGGGTCAGCCAAG  *Sac* I  R:GACAAAGCTTTCAGTCATCAGAAAGTCACCTATGAG  *Hind* III | 340 | 64 |
| pMIR-Report-3’-UTR-S2 | F:GATAGAGCTCGTCTTTCTCTGCAGTCCCCAGCC  *Sac* I  R:GACAAAGCTTGAAATGAGACAGTTAGTTTTTATTTATTCC  *Hind* III | 402 | 56 |
| pMIR-Report-3’-UTR-AS1 | F: GACAAAGCTTGCGGAGGCCGGGTCAGCCAAG  *Hind* III  R: GATAGAGCTCTCAGTCATCAGAAAGTCACCTATGAG  *Sac* I | 340 | 64 |
| pMIR-Report-3’-UTR-AS2 | F: GACAAAGCTTGTCTTTCTCTGCAGTCCCCAGCC  *Hind* III  R: GATAGAGCTCGAAATGAGACAGTTAGTTTTTATTTATTCC  *Sac* I | 402 | 56 |
| pMIR-Report-3’-UTR-M1 | F:CAAATGAGAAACAAAAC**CACA**AAGAGAAGGAACTGG  R:CCAGTTCCTTCTCTT**TGTG**GTTTTGTTTCTCATTTG | 340 | 55 |
| pMIR-Report-3’-UTR-M2 | F:GAATCTGGGCAGCAC**CACA**AAACCAACATTTCTTCAG  R:CTGAAGAAATGTTGGTTT**TGTG**GTGCTGCCCAGATTC | 402 | 55 |

Abbreviations: F, forward primer; R, reverse primer; and, bp, base pair. The mutated residues are in bold in primers for the generation of pMIR-Report-3’-UTR-M1F, pMIR-Report-3’-UTR-M2F, pMIR-Report-3’-UTR-MF, pMIR-Report-3’-UTR-M1 and pMIR-Report-3’-UTR-M2 constructs.

Table S6: *In silico* identification of microRNAs targeting the 3’-UTR of *MCPH1.*

| **MicroCosm** | **miRTAR** | **microRNA** | **miRDB** | **TargetScan** |
| --- | --- | --- | --- | --- |
| hsa-miR-101  hsa-miR-872  **hsa-miR-27a**  **hsa-miR-27b**  hsa-miR-367  hsa-miR-872  hsa-miR-643  hsa-miR-19b  hsa-let-7a  hsa-miR-181a  hsa-miR-593  hsa-miR-548 | hsa-miR-1278  hsa-miR-30a  hsa-miR-143  **hsa-miR-27a**  **hsa-miR-27b**  hsa-let-7a  hsa-let-7b  hsa-let-7c  hsa-miR-146a  hsa-miR-192  hsa-miR-139-5p | **hsa-miR-27a**  **hsa-miR-27b**  hsa-miR-145  hsa-miR-494  hsa-miR-33a  hsa-miR-33b  hsa-miR-599  hsa-miR-653  hsa-miR-381  hsa-miR-300  hsa-miR-599  hsa-miR-129-5p  hsa-let-7a  hsa-let-7b  hsa-let-7c | hsa-miR-532-5p  hsa-miR-616  hsa-miR-409-3p  hsa-miR-579  hsa-miR-1284  hsa-miR-607  **hsa-miR-27a**  **hsa-miR-27b**  hsa-miR-145  hsa-miR-139-5p  hsa-miR-942  hsa-miR-588  hsa-miR-361  hsa-miR-33b  hsa-miR-129-5p | hsa-miR-197  hsa-miR-450  hsa-miR-513  hsa-miR-128a  hsa-miR-128b  **hsa-miR-27a**  **hsa-miR-27b**  hsa-miR-412  hsa-miR-361  hsa-miR-224  hsa-miR-181a  hsa-miR-181b  hsa-miR-181c  hsa-miR-494  hsa-miR-340 |

**Table S7**: Mutations reported so far in the *MCPH1* gene.

| **SI.#** | **Mutation** | **Location** | **Nature of mutation** | **State of zygosity** | **Predicted effect on protein** | **Disease condition/**  **cell lines** | **Reference** |
| --- | --- | --- | --- | --- | --- | --- | --- |
| 1 | del exon 1-6 | Exon1-6 | Deletion | Homozygous | Results in truncated protein | MCPH | Darvish et al. (2010) |
| 2 | del exon 2-3 | Exon 2 & 3 | Deletion | Homozygous | Results in truncated protein | MCPH | Darvish et al. (2010) |
| 3 | c.74G>C(p.Ser25X) | Exon 2 | Nonsense | Homozygous | Results in truncated protein | MCPH | Jackson et al. (2002) |
| 4 | c.80C>G(p.Thr27Arg) | Exon 2 | Missense | Homozygous | Results in impaired function of N- terminal BRCT domain | MCPH | Trimborn et al. (2005) |
| 5 | c.147C>G (p.His49Gln) | Exon 3 | Missense | Homozygous | Results in impaired function of N- terminal BRCT domain | MCPH | Darvish et al. (2010) |
| 6 | del exon 3 | Exon 3 | Deletion | Homozygous | Results in truncated protein | MCPH | Darvish et al. (2010) |
| 7 | c.215C>T(p.Ser72Leu) | Exon 3 | Missense | Homozygous | Results in impaired function of N- terminal BRCT domain | MCPH | Darvish et al. (2010) |
| 8 | p.Trp75Arg | Exon 3 | Missense | Homozygous | Results in impaired function of N- terminal BRCT domain | MCPH | Ghani-Kakhki et al. (2012) |
| 9 | del exon 4 | Exon 4 | Deletion | Homozygous | Results in truncated protein | MCPH | Darvish et al. (2010) |
| 10 | c. 302C>G(p.S101X) | Exon 4 | Nonsense | Homozygous | Results in truncated protein | MCPH | Farooq et al. (2010) |
| 11 | c.321delA(p.Lys107fsX39) | Exon 4 | Frame-shift | Heterozygous | Results in truncated protein | Endometrial cancer; SCC084 & SCC131 cell lines | Bilbao et al. (2010); present study |
| 12 | c.436+1G>T | Intron 5 | Intronic | Homozygous | Results in truncated protein | MCPH | Darvish et al. (2010) |
| 13 | c.427_428insA(p.Thr143fsX5) | Exon 5 | Frame-shift | Homozygous | Results in truncated protein | PCC | Trimborn et al. (2004) |
| 14 | c.427delA(p.Thr143fsX3) | Exon 5 | Frame-shift | Heterozygous | Results in truncated protein | Endometrial cancer | Bilbao et al. (2010) |
| 15 | c.566_567insA (p.Asn189fsX15) | Exon 6 | Frame-shift | Homozygous | Results in truncated protein | MCPH | Darvish et al. (2010) |
| 16 | c.1179delG(p.Arg393fsX50) | Exon 8 | Frame-shift | Homozygous | Results in truncated protein | MCPH | Hussain et al. (2012) |
| 17 | c.1402delA(p.Thr468fsX32) | Exon 8 | Frame-shift | Heterozygous | Results in truncated protein | Endometrial cancer; SCC084 & SCC131 cell lines | Bilbao et al. (2010); present study |
| 18 | c.1561G>T(p.Glu521X) | Exon 8 | Nonsense | Homozygous | Results in truncated protein | OSCC | Present study |
| 19 | del exon 10 | Exon 10 | Deletion | Homozygous | Results in truncated protein | Breast cancer | Rai et al. (2006) |

Abbreviations: MCPH, autosomal recessive primary microcephaly; OSCC, oral squamous cell carcinoma; SCC084 and SCC131, oral cancer cell lines; and, PCC, premature chromosome condensation syndrome.

Table S8: Summary of the observed results across all the experiments.

| **Pt #** | **Real-time quantitative RT-PCR** | **IHC** | **LOH** | **Mutation** | **Promoter methylation** | **Western blotting** |
| --- | --- | --- | --- | --- | --- | --- |
| 5 |  |  | - |  |  |  |
| 8 |  |  | + |  |  |  |
| 9 |  |  | - |  |  |  |
| 11 |  |  | - |  |  |  |
| 14 |  |  | - |  |  |  |
| 13 |  |  | - |  |  |  |
| 12 |  |  | - |  |  |  |
| 15 |  |  | - |  |  |  |
| 17 |  |  | - |  |  |  |
| 18 | N |  | + |  | - |  |
| 19 |  |  | - |  |  |  |
| 20 |  |  | - |  |  |  |
| 21 | N |  | - |  | - |  |
| 22 | L |  |  |  | - |  |
| 26 |  |  | - |  |  |  |
| 27 |  |  | - |  |  |  |
| 30 |  |  | - |  |  |  |
| 34 |  |  | - |  |  |  |
| 38 | L |  | - |  | - |  |
| 39 |  |  | - |  |  |  |
| 40 |  |  | - |  | - |  |
| 47 | L |  | - |  | - |  |
| 44 |  |  | - |  |  |  |
| 49 | N |  | - |  | - |  |
| 50 |  |  | + |  | - |  |
| 52 |  |  | - |  | - |  |
| 53 |  |  |  |  | - |  |
| 54 |  |  | - |  |  |  |
| 55 |  |  | - |  |  |  |
| 56 | L |  | - |  | - |  |
| 57 | L |  |  |  | - |  |
| 59 |  |  | - |  | - |  |
| 60 |  |  | - |  | - |  |
| 62 | L |  | - |  | - | N |
| 63 | L |  | - | - | - | L |
| 65 |  |  | - |  |  |  |
| 66 | H |  | - |  | - |  |
| 67 | N |  | - |  | - |  |
| 68 | L |  | + | - | - | L |
| 69 | H |  | - |  |  |  |
| 70 |  |  | - |  |  |  |
| 71 |  |  | - |  |  |  |
| 72 |  |  | - |  |  |  |
| 73 |  |  | - |  |  |  |
| 74 |  |  | - |  |  |  |
| 75 |  |  | - |  |  |  |
| 76 | L |  | + | - | - |  |
| 77 |  |  | - |  |  |  |
| 79 | N |  | - |  | - |  |
| 80 | N |  | + | - | + |  |
| 83 |  |  | - |  |  |  |
| 92 | L |  |  |  | - | N |
| 95 | N |  | - |  | - |  |
| 101 | L |  | - | - |  |  |
| 108 | H |  | - | - |  |  |
| 109 | H |  | - |  |  | L |
| 110 | L | L | + | + | - |  |
| 113 | N |  | + |  | - |  |
| 114 |  |  | - |  |  |  |
| 115 | H | L | + |  |  |  |
| 116 | L | L | - |  | + |  |
| 121 | L |  |  |  | - |  |
| 125 |  | L | - |  |  |  |
| 127 |  | L |  |  |  |  |
| 128 | H | L |  |  |  | N |
| 133 | L | L | - |  | - |  |
| 135 | N | L |  |  | - |  |
| 139 | H | H | - |  |  |  |
| 140 | N | H | - |  |  | N |
| 143 |  | H | - | - |  |  |
| 144 | L | L | + |  | - |  |
| 155 | N |  | - |  | - | L |
| 156 |  | L | + |  | - |  |
| 158 |  | L |  |  |  |  |
| 157 |  | L | - |  |  |  |
| 159 |  | L | - | - |  |  |
| 165 |  |  | - |  |  |  |
| 166 |  |  | - |  |  |  |
| 174 |  |  | - |  |  |  |
| 175 |  | L | - |  | - |  |
| 177 | L | L | - |  | + |  |
| 180 |  |  | + |  | - |  |
| 181 | L | L | + | - | - |  |
| 182 |  |  | + |  |  |  |
| 183 |  |  | - |  |  | L |
| 191 |  |  | - |  |  | L |
| 192 |  |  | - |  |  |  |
| 194 |  | H |  |  |  |  |
| 202 | L | L | - | - | + |  |
| 206 | N | L |  | - |  |  |
| 212 | N | L | - | - |  |  |
| 214 | L | H |  | - | - |  |
| 219 | L | H | - | - | - |  |

Note that the data of the real-time quantitative RT-PCR is indicated according to 50%-150% cut-offs. Empty boxes indicate experiments not performed. Abbreviations:

-, not seen; +, seen; H, upregulated; L, downregulated; and, N, no change.
