# Supplementary material for: Primary Microcephaly Gene MCPH1 Shows Signatures of Tumor Suppressors and Is Regulated by miR-27a in Oral Squamous Cell Carcinoma
Source: PLoS One. 2013 Mar 5;8(3):e54643. doi: 10.1371/journal.pone.0054643 (PMC3589425; doi:10.1371/journal.pone.0054643)
Supplement: File S2 — Supporting multiple figures. (DOCX) [file pone.0054643.s002.docx]

**File S2:** Supporting multiple figures

**Primary microcephaly gene *MCPH1* shows signatures of tumor suppressors and is regulated by miR-27a in oral squamous cell carcinoma**

Thejaswini Venkatesh^1^, Mathighatta Nagaraj Nagashri^1^, Shivananda S. Swamy^2^, S.M. Azeem Mohiyuddin^3^, Kodaganur S. Gopinath^2^ and Arun Kumar^1^

^1^Department of Molecular Reproduction, Development and Genetics, Indian Institute of Science, Bangalore 560012, Karnataka, India. ^2^Department of Surgical Oncology, Bangalore Institute of Oncology, Bangalore 560027, Karnataka, India. ^3^Department of Otolaryngology and Head and Neck Surgery, R.L. Jalappa Hospital and Research Centre, Kolar 563101, Karnataka, India.

**Corresponding Author**

Prof. Arun Kumar

Department of Molecular Reproduction, Development and Genetics

Indian Institute of Science

Bangalore 560012

India

Email: karun@mrdg.iisc.ernet.in

Telephone: 91-80-2293 2998

Fax: 91-80-2360 0999

**Figure S1.** **LOH at the MCPH1 locus in pt# 110.** Phosphor image of the gel showing the LOH in the tumor at the marker D8S277. B and T denote constitutive blood and tumor DNA respectively. An arrow indicates LOH in the tumor DNA.


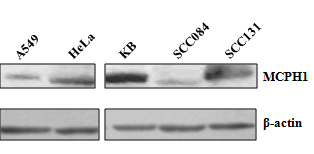


**Figure S2. MCPH1 protein levels in cancer cell lines.** SCC084 and SCC131 cells harboring the mutated *MCPH1* gene showed reduced levels of MCPH1 expression than KB cells with the wild-type *MCPH1*. A549 and HeLa cells with the wild-type *MCPH1* exhibited a lower expression of MCPH1 compared to KB cells.


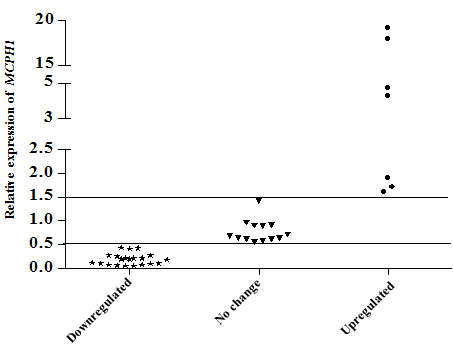


**Figure S3.** **The transcript levels of *MCPH1* in OSCC samples.** Note that the relative expression of *MCPH1* was downregulated in a majority of 21/41 (51.22%) tumor samples. The relative expression of *MCPH1* was upregulated in 7/41(17.07%) tumor samples and no change in its expression was seen in 13/41 (31.71%) of the tumor samples.

**
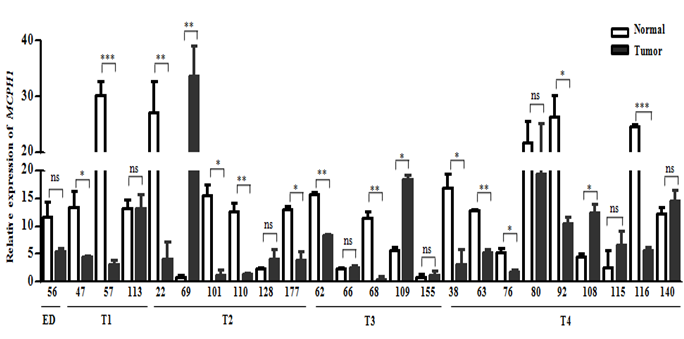
**

**Figure S4.** ***MCPH1* transcript levels in 24 matched OSCC samples.** Abbreviations: ns, statistically not significant; * , p<0.05; **, p<0.005; and, *** , p<0.001. Tumor samples are arranged according to their T classification (T1 to T4) or ED (epithelial dysplasia).


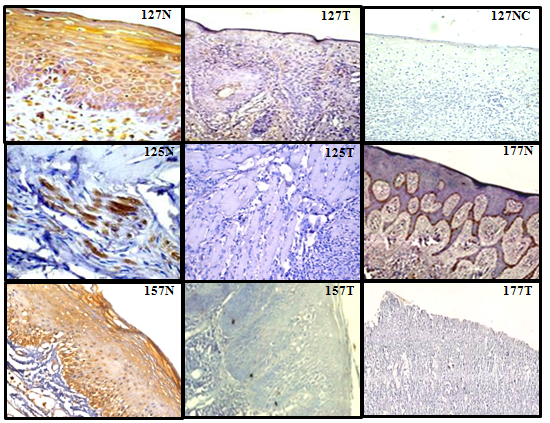


**Figure S5.** **MCPH1 expression in OSCC samples by immunohistochemistry.** Representative images are shown. Normal and tumor samples are marked as N and T respectively with their patient numbers. NC indicates staining without a primary antibody. Note downregulation (less staining) of MCPH1 in tumors compared to their matched normal tissues.

**
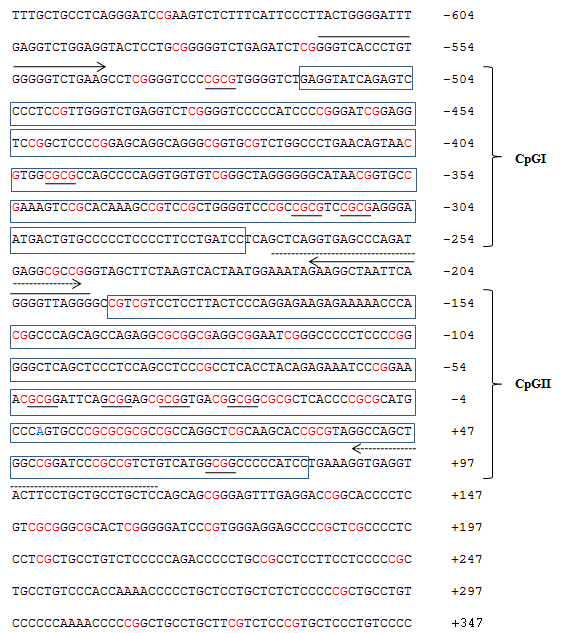
**

**Figure S6.** **Sequence of the *MCPH1* promoter with CpG islands.** The *MCPH1* promoter sequence retrieved from the Transcriptional Regulatory Element Database (TRED) is shown. CpG islands (CpGI and CpGII) are boxed and marked. Transcription start site (TSS) is marked in blue. Nucleotides are numbered with respect to the TSS. The CpG sites are in red. The *Bst* UI and *Aci* I restriction enzyme sites in CpGI and CpGII, respectively, are underlined. Continuous and dashed arrows mark the inner primers for the nested PCR of CpGI and CPGII respectively.

**
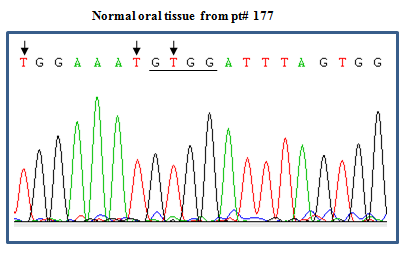
**


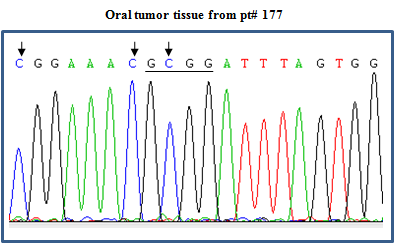


**Figure S7.** **Methylation of CpGII in OSCC.** Representative bisulfite sequencing chromatograms of a portion of CpGII in matched normal (N) and tumor (T) oral tissues from pt# 177. Arrows mark Ts and Cs at the CpG sites in normal and tumor tissues respectively. Note the retention of the *Aci* I site (underlined) in tumor and its loss in normal tissue after sodium bisulfite sequencing.


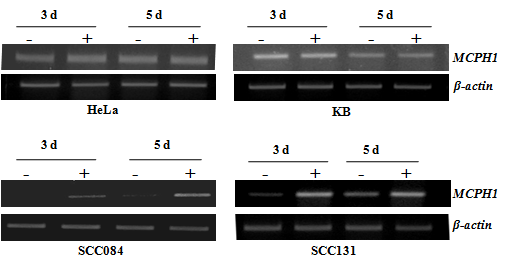


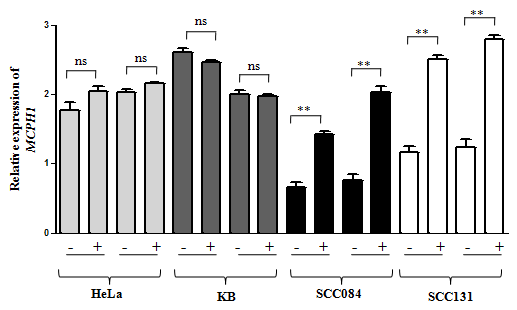


**Figure S8.** **Effect** **of the 2’-deoxy-5-azacytidine treatment on *MCPH1* expression in cell lines**. Upper and middle panels are representative agarose gel images showing levels of *MCPH1* transcripts following treatment of cell lines for 3 and 5 days. *ß-actin* was used for normalization. Note that the *MCPH1* levels were unchanged in HeLa and KB cells, whereas SCC084 and SCC131 cells showed upregulation of *MCPH1*, indicating methylation of its promoter. The lower panel is the quantitative representation of *MCPH1* expression normalised to *ß-actin* after the AZA treatment in cell lines. The values are the mean±SD of three independent experiments. Only the SCC084 and SCC131 cells exhibited significant upregulation of *MCPH1* after the treatment. Abbreviations: –, no treatment; +, treatment; d, days; ns, statistically not significant; and, **, p<0.005.


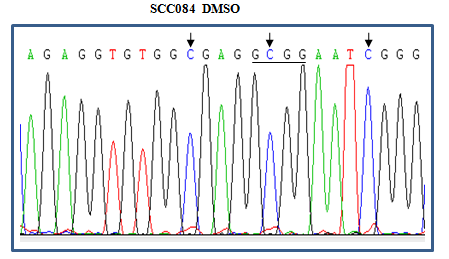


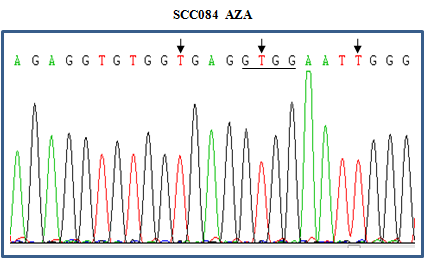


**Figure S9.** **Methylation of CpGII in SCC084.** Representative bisulfite sequencing chromatograms of a portion of CpGII in SCC084 cells before (vehicle control DMSO treated) and after the AZA treatment. Arrows mark Cs and Ts at the CpG sites before and after the AZA treatment. Note the conversion of C to T after the treatment. Also note the retention and the loss of an *Aci* I site (underlined) before and after the AZA treatment respectively. This suggests methylation of CpGII in SCC084 cells.


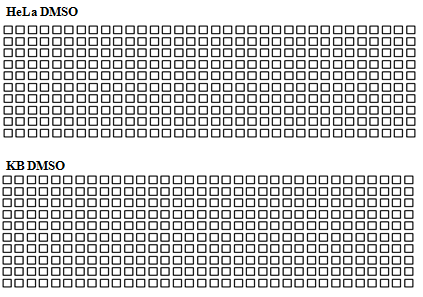


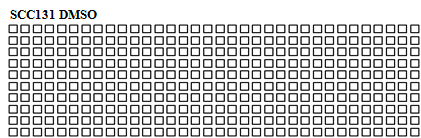


**Figure S10.** **Methylation status of CpGII in HeLa, KB and SCC131 cells.** Schematic representations of CpG sites in CpGII of bisulfite treated genomic DNA from HeLa, KB and SCC131 cells without AZA treatment (DMSO treated). Each row represents a TA clone. Unfilled squares represent unmethylated CpGs. Note the absence of methylation in CpGII in HeLa and KB cells as expected. Although the AZA treatment of SCC131 cells increased *MCPH1* levels, yet CpGII in this cell line was unmethylated, suggesting methylation of some other regions in the *MCPH1* promoter or some other alternative mechanism for its upregulation.


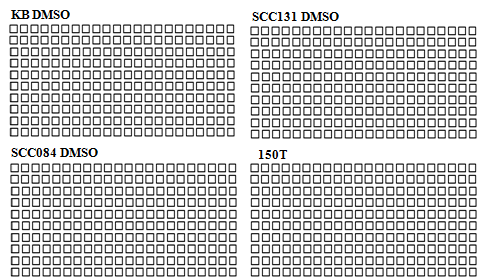


**Figure S11.** **Bisulfite** **sequencing of CpGI in oral cancer cell lines and an OSCC sample.** Schematic representation of the methylation status of CpG sites in CpGI in bisulfite treated genomic DNA from KB, SCC084 and SCC131 cells without AZA treatment (DMSO treated), and an OSCC sample (150T). Each row represents a TA clone. The unfilled squares represent unmethylated CpGs. Note the absence of methylation.

**Figure S12.** **The soft agar colony formation assay.** Note that the number of colonies per field is significantly reduced in MCPH1 overexpressing clones B1 and B9 in comparison to vector clones V2 and V4, and KB cells. The values are the mean±SD of three independent experiments. Abbreviations: ns, statistically not significant; and, *** , p<0.001.


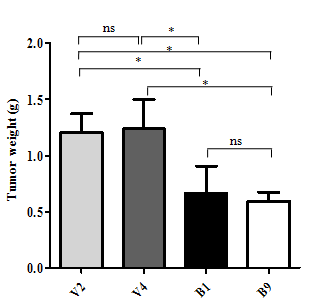


**Figure S13. Graphical representation of tumor weights of stable clones in nude mice.** Note that the tumor weights are significantly reduced in MCPH1 overexpressing clones B1 and B9 in comparison to vector clones V2 and V4. The values are the mean±SD of xenografts from five animals. Abbreviations: ns, no significance; and, * , p<0.05.


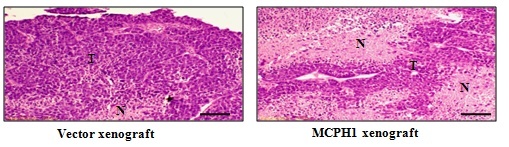


**Figure S14.** **Representative images of the hemotoxylin and eosin stained xenografts.** Note a higher number of nectrotic regions in the xenograft from B9 cells in comparison to the vector containing xenograft (V2 cells). Abbreviations: N, necrotic regions; and, T, tumor regions. Scale = 100 µm.


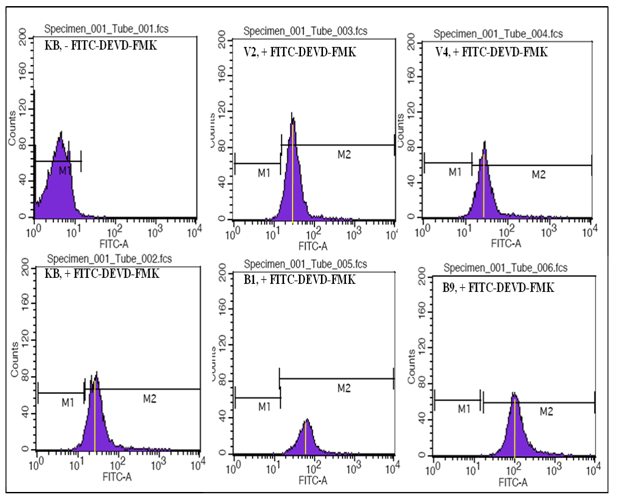


**Figure S15.** **MCPH1 overexpression increases apoptosis.** Cells in apoptosis were quantitated by measuring the activity of CASP3 by flow cytometry. Note that the right shifts in the FITC channel of B1 and B9 cells in comparison to that of KB, V2 and V4 cells indicate a higher CASP3 activity. Abbreviations: KB, parental cell line; V2 and V4, empty vector clones; B1 and B9, MCPH1 overexpressing clones; - FITC-DEVD-FMK, no incubation with FITC-conjugated CASP3 inhibitor; and, + FITC-DEVD-FMK, incubation with FITC-conjugated CASP3 inhibitor.


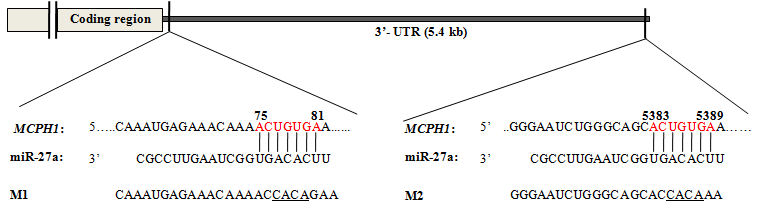


**Figure S16.** **miR-27a binding sites in the *MCPH1* 3’-UTR.** Sequence alignment of two seed regions (SDR1, 75 nt to 81 nt; and, SDR2, 5383 nt to 5389 nt) of the *MCPH1* 3’-UTR with miR-27a. SDR1 and SDR2 sequences are in red. M1 and M2 represent sequences in constructs with mutations in SDR1 and SDR2 respectively. Mutated nucleotide residues are underlined.


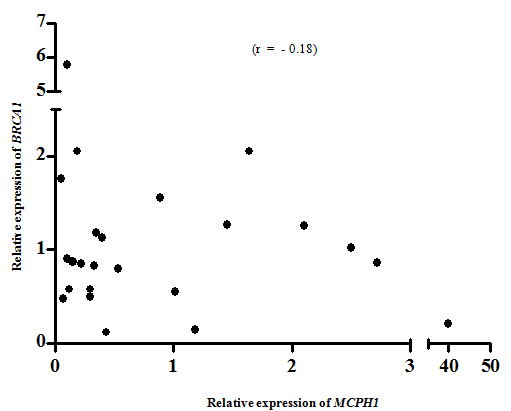


**Figure S17. No correlation between the levels of *MCPH1* and *BRCA1* transcripts in OSCC.** Note the Pearson’s coefficient r is near to zero, showing no significant correlation. The values shown are the mean of three independent experiments.


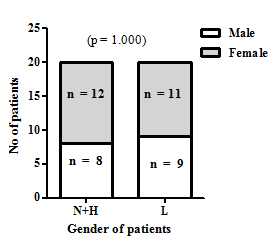

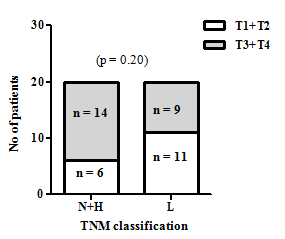


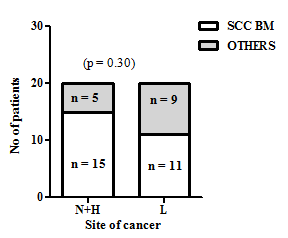

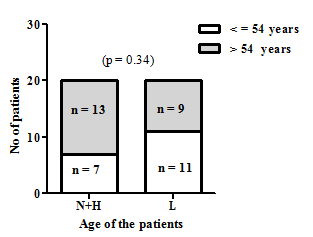


**Figure S18.** **Correlation of the *MCPH1* transcript levels with clinico-pathological parameters.** *MCPH1* transcript levels showed no correlation with gender of the patients, T stages of the tumors, age of the patients and site of the tumors by Fischer’s exact test. T1, T2, T3 and T4 represent the different stages of tumors. Abbreviations: SCC BM, squamous cell carcinoma of buccal mucosa; Others, sites of tumors from gingiva, alveolus, floor and angle of the mouth, lip and retro molar trigone; L, tumor samples with downregulated *MCPH1* transcript; H, tumor samples with upregulated *MCPH1* transcript; and, N, no change in the level of *MCPH1* transcript between normal and tumor tissues.


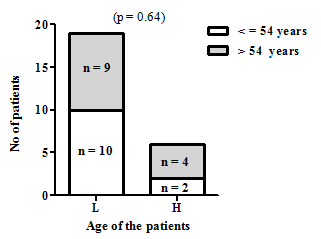

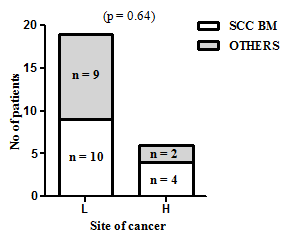

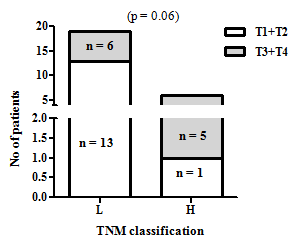

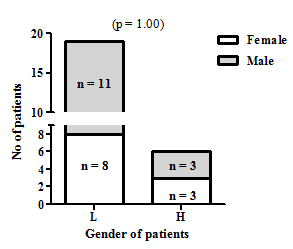


**Figure S19. Correlation of the MCPH1 protein levels with clinico-pathological parameters.** MCPH1 protein levels showed no correlation to gender of the patients, T stages of the tumors, age of the patients and site of the tumors by Fischer’s exact test. Abbreviations as in Figure S18.
